# Crystal Structure of an Ammonia-Permeable Aquaporin

**DOI:** 10.1371/journal.pbio.1002411

**Published:** 2016-03-30

**Authors:** Andreas Kirscht, Shreyas S. Kaptan, Gerd Patrick Bienert, François Chaumont, Poul Nissen, Bert L. de Groot, Per Kjellbom, Pontus Gourdon, Urban Johanson

**Affiliations:** 1 Department of Biochemistry and Structural Biology, Center for Molecular Protein Science, Lund University, Lund, Sweden; 2 The Max Planck Institute for Biophysical Chemistry, Computational Biomolecular Dynamics Group, Göttingen, Germany; 3 Institut des Sciences de la Vie, Université catholique de Louvain, Louvain-la-Neuve, Belgium; 4 IPK—Leibniz Institute of Plant Genetics and Crop Plant Research Department of Physiology and Cell Biology, Gatersleben, Germany; 5 Danish Research Institute of Translational Neuroscience–DANDRITE, Nordic-EMBL Partnership for Molecular Medicine, Department of Molecular Biology and Genetics, Aarhus University, Aarhus C, Denmark; University of Zurich, SWITZERLAND

## Abstract

Aquaporins of the TIP subfamily (Tonoplast Intrinsic Proteins) have been suggested to facilitate permeation of water and ammonia across the vacuolar membrane of plants, allowing the vacuole to efficiently sequester ammonium ions and counteract cytosolic fluctuations of ammonia. Here, we report the structure determined at 1.18 Å resolution from twinned crystals of *Arabidopsis thaliana* aquaporin *At*TIP2;1 and confirm water and ammonia permeability of the purified protein reconstituted in proteoliposomes as further substantiated by molecular dynamics simulations. The structure of *At*TIP2;1 reveals an extended selectivity filter with the conserved arginine of the filter adopting a unique unpredicted position. The relatively wide pore and the polar nature of the selectivity filter clarify the ammonia permeability. By mutational studies, we show that the identified determinants in the extended selectivity filter region are sufficient to convert a strictly water-specific human aquaporin into an *At*TIP2;1-like ammonia channel. A flexible histidine and a novel water-filled side pore are speculated to deprotonate ammonium ions, thereby possibly increasing permeation of ammonia. The molecular understanding of how aquaporins facilitate ammonia flux across membranes could potentially be used to modulate ammonia losses over the plasma membrane to the atmosphere, e.g., during photorespiration, and thereby to modify the nitrogen use efficiency of plants.

## Abstract 2

The structure of an ammonia channel from plants extends our understanding of substrate specificity in different types of aquaporins and reveals an intriguing side pore that raises new questions.

## Author Summary

Ammonia is a central molecule in nitrogen metabolism. Aquaporins are integral membrane proteins that form channels that accelerate the passive permeation of small polar uncharged molecules, like water and ammonia, across lipid membranes of the cell. Structural information of ammonia-permeable aquaporins has been lacking. Here, we report a high-resolution structure of the ammonia-permeable aquaporin *At*TIP2;1 and explore it by functional assays of mutants and by molecular dynamics simulations. Our data uncover unexpected features of the substrate selectivity filter, including a conserved arginine in a new orientation that is stabilized by interactions to a histidine that is linked to ammonia specificity. An additional histidine in a different part of *At*TIP2;1 fortifies the position of the arginine and interacts directly with the substrate in the channel. This histidine is therefore included in an extended selectivity filter, which should prompt a reinterpretation of the determinants of specificity in all types of aquaporins. We speculate that an intriguing water-filled side pore, next to the substrate-binding histidine, participates in deprotonating ammonium ions, which could increase the net permeation of ammonia. Understanding the principles of ammonia permeability may, in the future, allow us to modulate the passage of ammonia and generate crops with higher nitrogen-use efficiency.

## Introduction

Nitrogen is a macronutrient that is often limiting for plant growth. Hence, efficient channeling and storage of ammonia, a central molecule in nitrogen metabolism, is of fundamental importance. Tonoplast Intrinsic Proteins (TIPs) belonging to the Major Intrinsic Protein family, also known as the aquaporin (AQP) superfamily, have been shown to conduct both water [1] and ammonia [2–4]. TIPs are present in all land plants, but whereas primitive plants like mosses only have one type of TIP (TIP6), five specialized subgroups (TIP1‒5) have evolved in higher plants [5]. TIPs may constitute up to 40% of the protein in the vacuolar membrane, i.e., the tonoplast [6], and have been suggested to enhance nitrogen uptake efficiency and detoxification by acid entrapment of ammonium ions in vacuoles [3]. Furthermore, TIP-mediated increase of ammonia permeability was proposed to play a role in remobilization of vacuolar ammonia during nitrogen starvation [2] and in reallocation of nitrogen at senescence [7]. Recently, TIPs were included in a revised model of futile cycling under high ammonia conditions [8]. Sequence similarities to TIPs are observed in mammalian AQP8s [9], which are also ammonia-permeable [10] and have been implicated in pathological conditions like hyperammonemia and hepatic encephalopathy [11].

**Fig 1:**
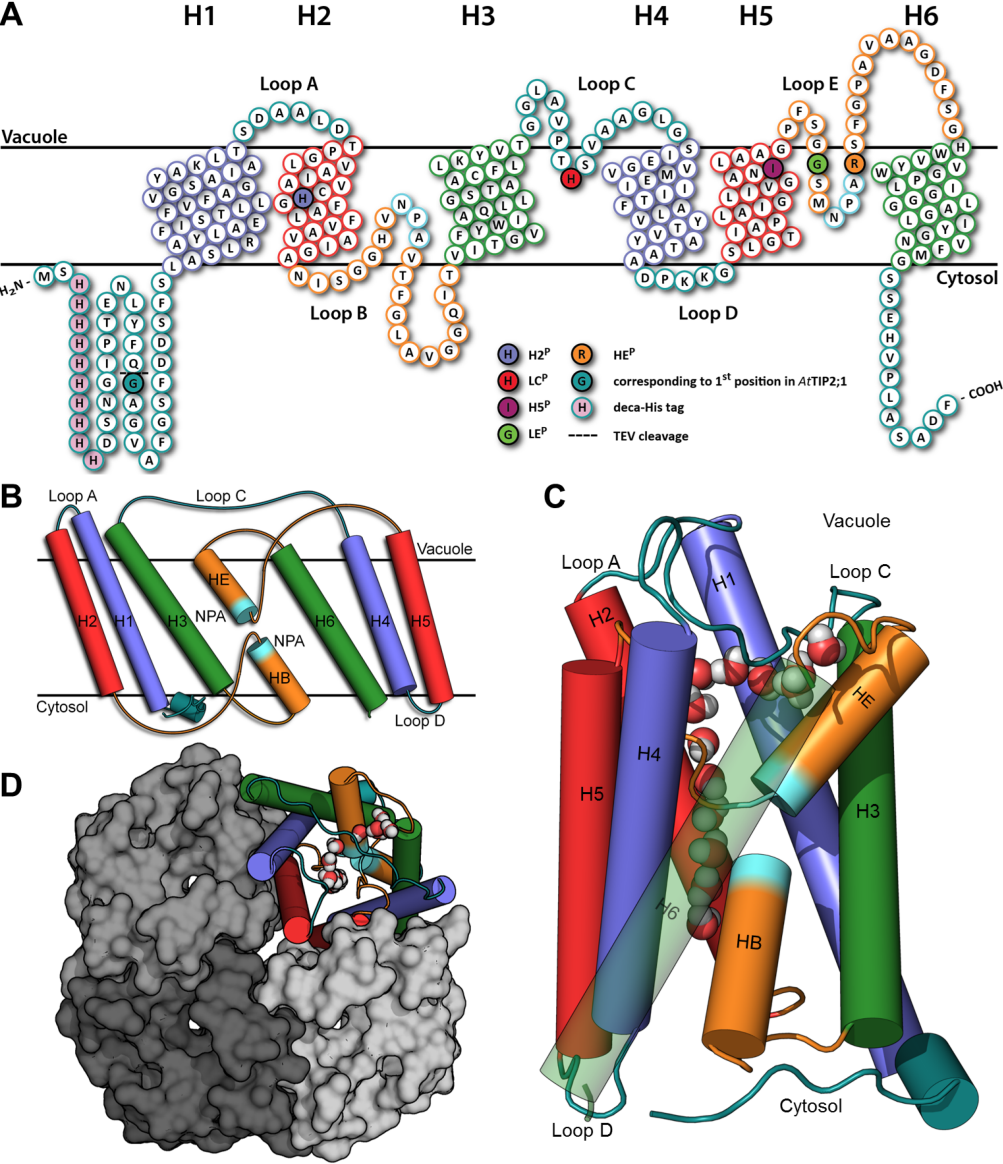
Topology and structure of *At*TIP2;1. (A) Topology plot showing membrane-spanning helices (H1–H6) and intervening loops (A–E). Homologous regions in the internal repeat are indicated by colors. The five positions of an extended selectivity filter are marked according to the key within the figure. The glycine (Gly 1) corresponding to the initiator methionine in *At*TIP2;1 is shaded in dark cyan next to a dashed line representing the TEV cleavage site, and the N-terminal deca-His tag is shaded in purple. (B) Two short helices (HB and HE) in loop B and E, connected via conserved NPA-motifs, form a seventh transmembrane segment. All membrane-spanning segments are tilted in the membrane, but it is most accentuated in helices H3, H6, HB, and HE facing the lipid bilayer. (C) Eight water molecules form a single file in the main pore of the monomer, connecting the cytosolic and vacuolar vestibules. At the top right, five additional water molecules are seen in a side pore underneath loop C. (D) *At*TIP2;1 tetramer viewed from the vacuolar side. Monomers are shown in surface representation and in the cartoon representation used in (B) and (C).

Crystal structures have established that AQPs are homotetramers, where each of the monomers holds a functional pore created by six membrane-spanning helices (helix 1‒helix 6), five connecting loops (loop A‒loop E), and two shorter helices (helix B and helix E; Fig 1), both displaying the AQP-signature motif Asn-Pro-Ala (NPA) [12–16]. Helices B and E connect at the NPA motifs in the middle of the membrane, thus forming a bipartite transmembrane segment. Different AQP isoforms facilitate permeation of a variety of small uncharged polar molecules, while protons are efficiently excluded from the pore in part by the positive charge, which is focused at the NPA region by the macro dipoles of the short helices [17]. Substrate specificity is thought to be achieved by the aromatic/arginine selectivity filter [18], which has been defined as four residues located at the noncytosolic end of the pore [19]. Of these residues, an arginine is conserved in the short helix E of most AQPs and contributes to the exclusion of protons [20], whereas a histidine in helix 5 is associated with water specificity [13]. AQPs permeable to ammonia and water called aquaammoniaporins, including the human *Hs*AQP8, typically lack the histidine in helix 5 but instead have a histidine in helix 2 [2–4]. However, all previously published AQP structures represent either water-specific channels (true AQPs) or the water- and glycerol-conducting aquaglyceroporins, so a further understanding of the structural features that confer ammonia selectivity has been missing. To close this gap in knowledge, we set out to crystallize the aquaammoniaporin *At*TIP2;1 from *A*. *thaliana*. Here, we present the crystal structure of *At*TIP2;1 determined at atomic resolution (1.18 Å with partial twinning). Combined with molecular dynamics (MD) simulations and functional studies of mutants, the structure provides new insight into the molecular basis of substrate selectivity in the AQP superfamily.

## Results

### Overall Structure of *At*TIP2;1

**Fig 2:**
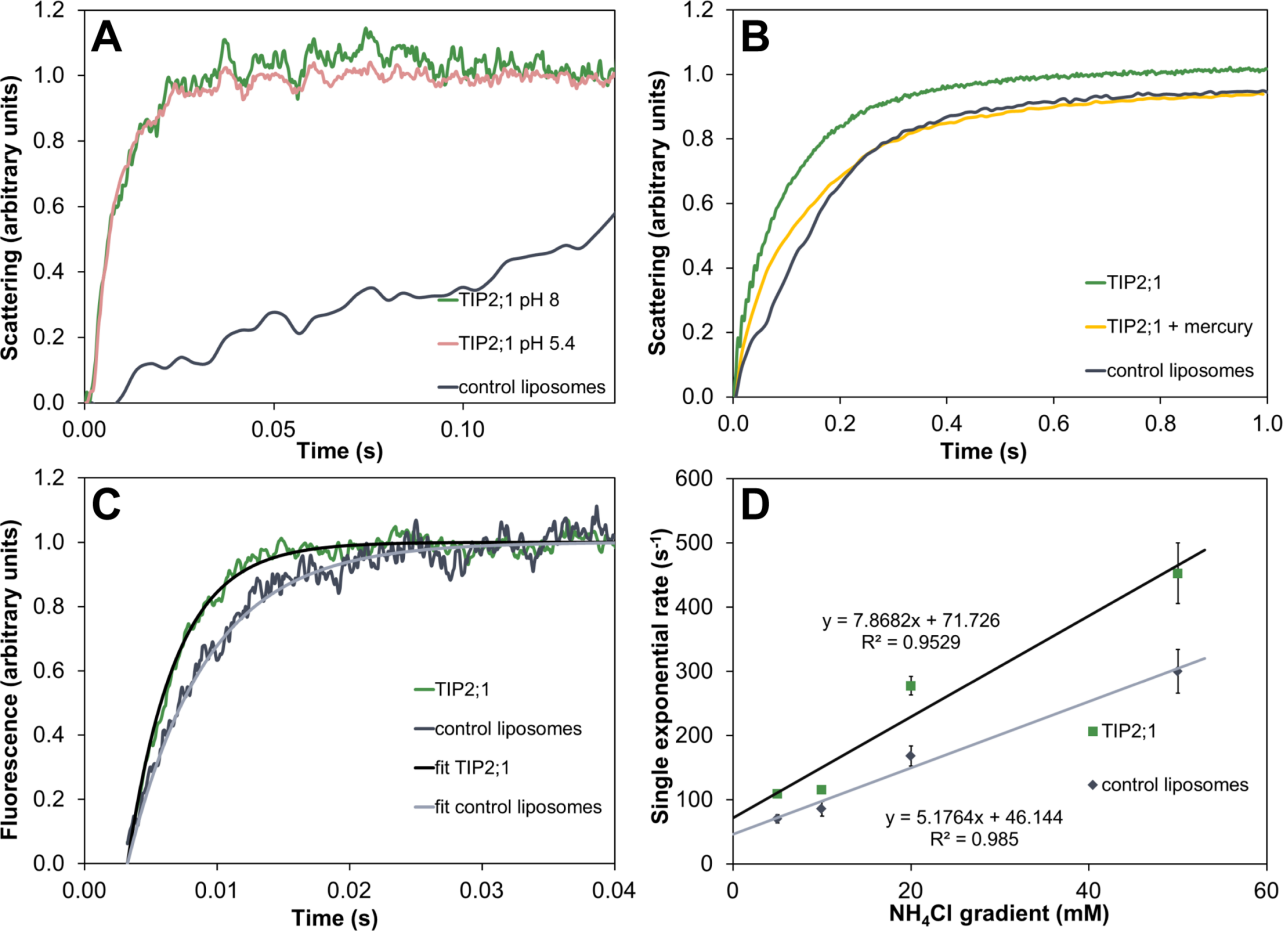
Functional assays. (A) Water permeability of liposomes with and without inserted *At*TIP2;1 measured at different pH values. Stopped-flow experiments with 100 mM hyperosmolar shift present high protein-facilitated conductivities at cytosolic and vacuolar pH. Relative single exponential rate constants of ca. 110 s^−1^ at LPR 30 demonstrate the ability to sustain a highly water-permeable vacuole. (B) Water permeability of purified *At*TIP2;1 (LPR 50) is inhibited by mercury as previously shown in oocytes [21]. (C) Ammonia uptake monitored by increased internal fluorescence after exchange of 20 mM NaCl with 20 mM NH_4_Cl, corresponding to an initial NH_3_ gradient of 4.5 μM. Proteoliposomes with *At*TIP2;1 (green) show higher permeability than equally-sized control liposomes (grey). Fitted curves yielding single exponential rates are also shown. (D) Summary of rates at different ammonia gradients. Equations and correlation coefficients are given for linear fitting of averaged single exponential rates as a function of total initial concentration gradient of NH_3_/NH_4_^+^. Error bars represent the standard deviation of the rate within a set of approximately ten stopped-flow recordings per liposome sample. The underlying data of panels A–D can be found in S1 Data.

Heterologously expressed *At*TIP2;1 yielded up to 1.1 mg of purified and concentrated protein per g of wet *Pichia pastoris* cells. Purified *At*TIP2;1 was verified as a functional water channel, inhibited by mercury, and also permeable to ammonia (Fig 2). *At*TIP2;1, solubilized by n-octyl-β-D-glucoside, was crystallized at pH 5.0 and the structure determined at 1.18 Å resolution (Table 1). In the reported structure, 238 amino acid residues are resolved, and only the N-terminal tag and 12 native residues at the C-terminus are not included in the model. In contrast to other AQPs (e.g., *So*PIP2;1 [14], *Hs*AQP5 [15]), neither loops nor the resolved parts of terminal regions overlap with neighboring monomers in the tetramer. Loop A and loop D fold back on their own subunit and the N- and C-terminal regions meet at the outer edge of the cytoplasmic vestibule without restricting the pore (Fig 1C and 1D). Accordingly, the structure of *At*TIP2;1 constitutes an open channel where the cytosolic and vacuolar vestibules are connected by a pore lining eight water molecules in a single file.

**Table 1:** Crystallographic data and refinement statistics.

Data collection	
Space group	*I* 4
Cell dimensions	
*a*, *b*, *c* (Å) / *a*, *b*, *γ* (°)	89.3, 89.3, 82.5 / 90, 90, 90
Resolution (Å)	44.6–1.18 (1.22–1.18)*
*R*_merge_ (%)	7.46 (117)*
*I*/σ*_I_*	12.65 (1.63)*
CC_1/2_	1.00 (0.35)*
Completeness (%)/Redundancy	99.8 (98.0) / 13.2 (10.8)*
Refinement	
Resolution (Å)	44.6–1.18 (1.21–1.18)*
No. reflections	106,000 (10,267)*
Twin operator/Twin fraction (%)	k, h, -l / 40.7
*R*_work_ / *R*_free_ (%)	10.2 / 11.2
No. atoms	3,511
Protein (nonhydrogen)/Water	1,688 / 131
B-factors (Å^2^)	16.1
Protein / Water	15.0 / 29.6
R.m.s. deviations	
Bond lengths (Å) / angles (°)	0.011 / 1.36
Ramachandran outliers (%)	0.4
Ramachandran favored (%)	98.0
Clashscore	0.00

*Highest resolution shell is shown in parenthesis.

### An Extended Selectivity Filter

**Fig 3:**
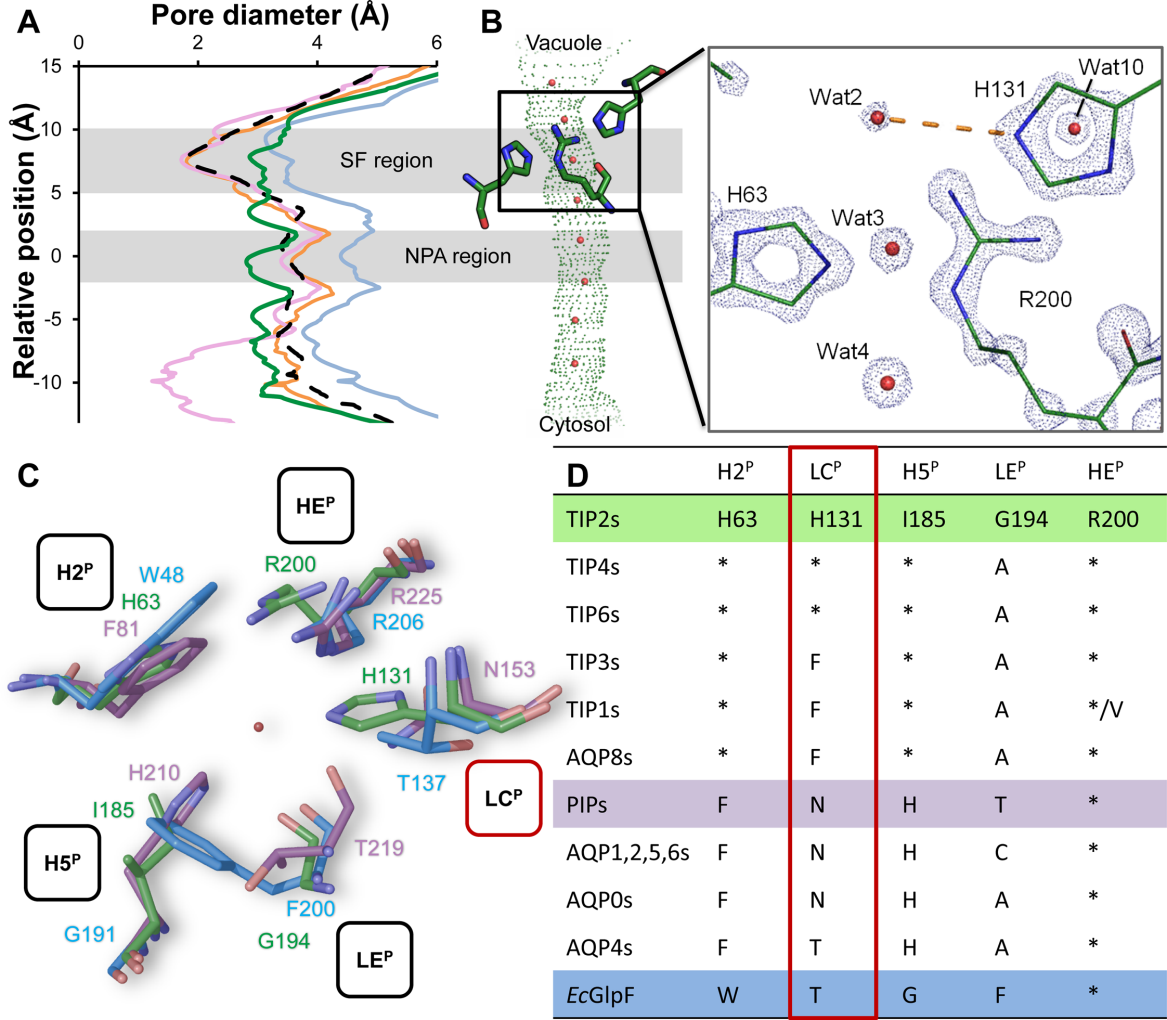
Pore diameter and the extended selectivity filter. (A) Individual profiles of *At*TIP2;1 (green), glycerol-permeable *Ec*GlpF (blue), water-specific *So*PIP2;1 (closed conformation; purple), and *Hs*AQP4 (orange), as well as average diameter of five other open water-specific AQP structures (dashed line). Protein Data Bank IDs are provided in S1 Table. NPA region and selectivity filter (SF) indicated by shading. In contrast to previously reported structures of AQPs, where the SF region constitutes the most narrow part of the channel, the pore diameter of *At*TIP2;1 is more uniform throughout the channel. (B) Graphic representation of a side view of the *At*TIP2;1 pore aligned with (A). The selectivity filter is highlighted by stick representation of residues in positions H2^P^, HE^P^, and LC^P^ (left to right). Nondisplayed residues at positions H5^P^ and LE^P^ are located in front of the visual plane. Close-up depicts electron density at 4σ. The high resolution of the structure makes it possible to pinpoint the nitrogen atoms in imidazole rings of histidines. The Nε of LC^P^-His 131 forms a hydrogen bond (dashed yellow line) to a water molecule (Wat2) in the pore. (C) Vacuolar (top view of *At*TIP2;1) and corresponding extracellular view (*So*PIP2;1 and *Ec*GlpF) on the amino acid residues at the five positions (H2^P^, LC^P^, H5^P^, LE^P^, and HE^P^) comprising the extended selectivity filter of the pore. *At*TIP2;1 (green) is compared to the water-specific *So*PIP2;1 (purple) and the glycerol-permeable *Ec*GlpF (blue). In *At*TIP2;1, histidines at H2^P^ and LC^P^ stabilize the arginine (Arg 200) at HE^P^ in a novel orientation, which is clearly different from its positioning in structures of water-specific and glycerol-permeable AQPs. The spatial orientation of the backbone carbonyls at position LE^P^ is similar in *At*TIP2;1 and *Ec*GlpF, whereas it deviates in the water-specific *So*PIP2;1. The Ile 185 at H5^P^ of *At*TIP2;1 results in a wider SF region compared to water-specific AQPs that have a histidine at this position. (D) The conservation of residues in the extended selectivity filter displayed in (C). The LC^P^ position that extends the selectivity filter is boxed in red. Plant TIPs and mammalian AQP8s are similar and distinctly different from water-specific AQPs in animals and plants (PIPs, plasma membrane intrinsic proteins), as well as the glycerol channel GlpF. Conservation patterns suggest a similar orientation of the conserved arginine at position HE^P^ of the selectivity filter of all TIPs and AQP8s, and furthermore that individual subgroups of TIPs and water-specific AQPs might have evolved specialized substrate profiles (details in S2 Table). Asterisk denotes identity to TIP2s, and colors highlight selectivity filters shown in (C).

Interestingly, the pore diameter of *At*TIP2;1 at the NPA region is smaller than in other AQPs, and it remains constant at around 3 Å throughout the pore (Fig 3A and 3B). This is unusual, since in other structures of open AQPs, the aromatic/arginine selectivity filter constitutes the narrowest part of the pore. As mentioned earlier, amino acid residues at the four positions of the pore selectivity filter in helix 2, helix 5, loop E, and helix E (specifically denoted H2^P^, H5^P^, LE^P^, and HE^P^) are thought to determine the substrate specificity (Figs 1A and 3C). In line with this, TIP2s deviate from other AQPs (Fig 3D), and as expected from mutational studies and modeling [2,22], the wider selectivity filter is mainly due to an isoleucine (Ile 185) at position H5^P^ in helix 5, replacing a histidine that is conserved in the water-specific AQPs. However, the most striking feature of the *At*TIP2;1 selectivity filter arises from an unpredicted positioning of the arginine at HE^P^ in helix E (Arg 200), a conserved residue in nearly all AQPs. In *At*TIP2;1, the arginine side chain is pushed to the side of the pore by a histidine located in loop C (His 131), which now appears as a fifth residue (LC^P^) of an extended selectivity filter. The novel position of the arginine is further stabilized by a hydrogen bond to the histidine (His 63) at position H2^P^ in helix 2, which occupies essentially the same space as corresponding aromatic residues of water and glycerol channels (e.g., Phe 81 in *So*PIP2;1 [14], Trp 48 in *Ec*GlpF [12]) without direct effects on the pore aperture. The close interaction with Arg 200 at position HE^P^ in helix E suggests a shift in the p*K*_*a*_ of His 63 at position H2^P^, which is likely to stay unprotonated also in the acidic environment of the vacuole. In contrast to His 63, the additional His 131 at position LC^P^ in loop C points to the center of the pore and forms a hydrogen bond to a pore-water (Wat 2; Fig 3B). Hence, *At*TIP2;1 represents the first AQP structure where a residue in loop C (His 131) directly participates in interactions with the substrate in the selectivity region, defining an extended selectivity filter with five positions. The histidine residue at position H2^P^ in helix 2 is conserved in all TIPs, whereas the histidine at position LC^P^ in loop C is only maintained in some types of TIPs, including the TIP2 isoforms, and appears to have been replaced by phenylalanine in a common ancestor of TIP1s and TIP3s (Fig 3D) [5]. A phenylalanine at position LC^P^ in loop C is also capable of sterically directing the arginine at position HE^P^ in helix E to the side of the pore, but provides a more hydrophobic environment at the selectivity filter. Worth noting, similar to TIP3s, the mammalian AQP8s [9,10] also possess a histidine at position H2^P^ in helix 2, lack a conserved histidine in loop C, and can be aligned with a phenylalanine at position LC^P^ in loop C (Fig 3D). Thus, a histidine at position H2^P^ in helix 2 and an aromatic residue at position LC^P^ in loop C seem to be a common feature among ammonia-permeable AQPs both in plants and animals. This suggests that the derived phenylalanine at LC^P^ in loop C of some plant TIPs, which supports ammonia permeability without the ability to form hydrogen bonds to the substrate, reflects an adaptation to a different milieu, e.g., regarding pH or alternatively altered requirements on permeation rate and selectivity.

### General Importance of Loop C in AQPs

Measurements of absolute water permeabilities [23] and relative permeabilities to other substrates [24] reveal large variations among water-specific AQPs. For example, mammalian AQP4 has a 4-fold higher single channel water permeability compared to AQP1, although they share identical or similar residues at the four canonical positions of the selectivity filter. Our structure led us to re-examine the amino acid residue at position LE^P^ in loop E, which contributes with its carbonyl to the selectivity region. Comparison of *At*TIP2;1 with other AQP structures identifies two spatial groups (I and II) of the carbonyls from residues at position LE^P^ in loop E as illustrated in Supporting Information (S1 Fig; top view in Fig 3C). *At*TIP2;1 groups together with aquaglyceroporins and the water-permeable human AQP4 (group I), whereas the majority of water-specific AQPs constitutes group II due to an asparagine at position LC^P^ in loop C (Fig 3D), which can form a hydrogen bond to the backbone carbonyl of the amino acid residue at position LE^P^. This group II-specific interaction directs the LE^P^ carbonyl to the center of the pore and quasiparallel to the membrane. In this conformation, the peptide bonds of this and the two preceding residues are contorted. In contrast, the AQPs lacking an asparagine at position LC^P^ place the backbone carbonyl of the amino acid residue at position LE^P^ (Gly 194 in *At*TIP2;1)—and preceding residues of loop E—in a more relaxed conformation, resembling that of a corresponding carbonyl (Gly 80) in the quasisymmetry-related loop B (Fig 1A). The variation of the carbonyls of the amino acid residues at position LE^P^ is spatially small and hence has little effect on the pore diameter per se, but it significantly affects hydrogen bonding to substrates. Thus, the identity of the amino acid residue at position LC^P^ in loop C appears to be relevant for substrate specificity, not only in ammonia permeable AQPs but also in water-specific isoforms.

### Ammonia Specificity

**Fig 4:**
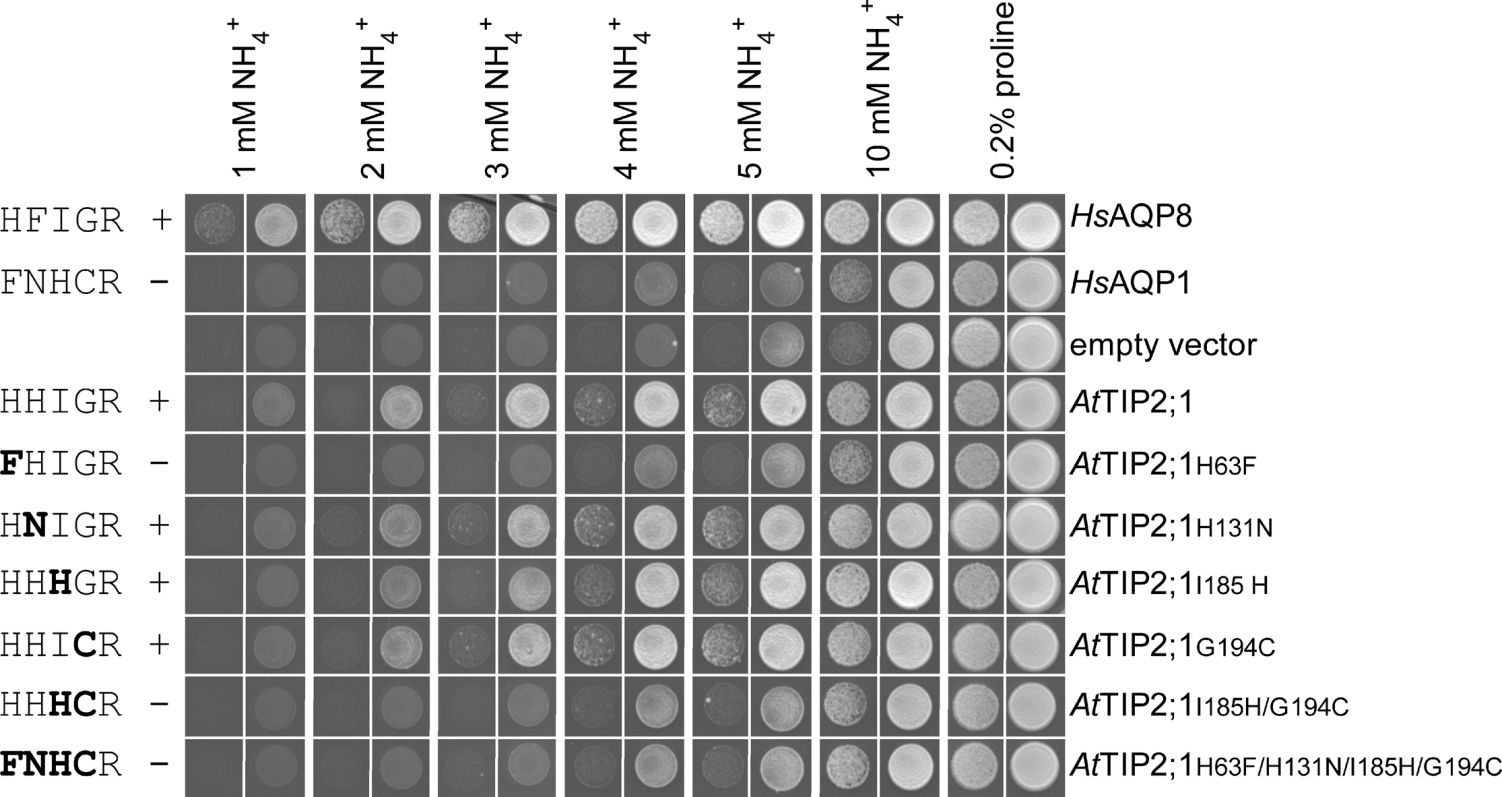
Growth complementation of ammonium uptake-defective yeast strain by mutants of *At*TIP2;1. The 31019b yeast strain (*Δmep1–3*) was transformed with the empty vector pYeDP60u or with pYeDP60u-carrying cDNA encoding the positive controls *Hs*AQP8 or *Hs*AQP1, or *At*TIP2;1 or its mutants. The five amino acid residues of the extended selectivity filter in each construct are indicated in one letter code to the left in the order H2^P^, LC^P^, H5^P^, LE^P^, and HE^P^, showing substitutions in bold. Complementation and failure to complement are indicated by + and −, respectively. Transformants were spotted at an OD_600_ of 1 (right column) and 0.01 (left column) on plates containing 0.2% proline or the indicated concentrations of ammonium as a sole nitrogen source and growth was recorded after 13 d at 28°C. Each panel showing growth at a specific concentration is compiled by individual pictures of each spot taken from a distinct growth condition. All single pictures were treated in the same manner.

To investigate the contribution of the amino acid residues in the extended selectivity filter to substrate specificity, we analyzed the permeability of the water-specific human AQP1 (*Hs*AQP1) and *At*TIP2;1 using in vivo and in vitro assays. Both these channels have the conserved arginine at position HE^P^ of the selectivity filter but, as established by our structure, the spatial location of its side chain relative to the pore differs. Expectedly, substituting all four deviating residues of the extended selectivity filter in a quadruple mutant of *At*TIP2;1 to the corresponding residues of *Hs*AQP1 abolished complementation in a yeast growth assay probing for ammonia permeability (Fig 4). Single point mutations at each of the four positions specify that all of the individual substitutions except the exchange of histidine for phenylalanine at H2^P^ are compatible with ammonia permeability in *At*TIP2;1. The incompatibility of the phenylalanine may be explained by its different electrostatics and slightly larger size, which would be in conflict with the orientation of the arginine at position HE^P^ in the selectivity filter of *At*TIP2;1. The spatial orientation of the arginine at position HE^P^ in *At*TIP2;1 is constrained by loop C and the histidine at position LC^P^, hence the introduced phenylalanine at H2^P^ is likely to adopt an alternative position where it occludes the pore. The compatibility of the three other single mutations may at first appearance be explained by their polar nature, allowing formation of hydrogen bonds to ammonia. However, as mentioned above, at position LE^P^, the hydrogen bond to the substrate is offered by the backbone carbonyl rather than the side chain. Interestingly, a double mutation in helix 5 (position H5^P^) and loop E (position LE^P^) of the selectivity filter failed to demonstrate ammonia permeability in the growth assay. This may indicate that a small and flexible residue at position LE^P^ of *At*TIP2;1 is required to allow its carbonyl to interact with the substrate in the presence of a histidine at position H5^P^.

**Fig 5:**
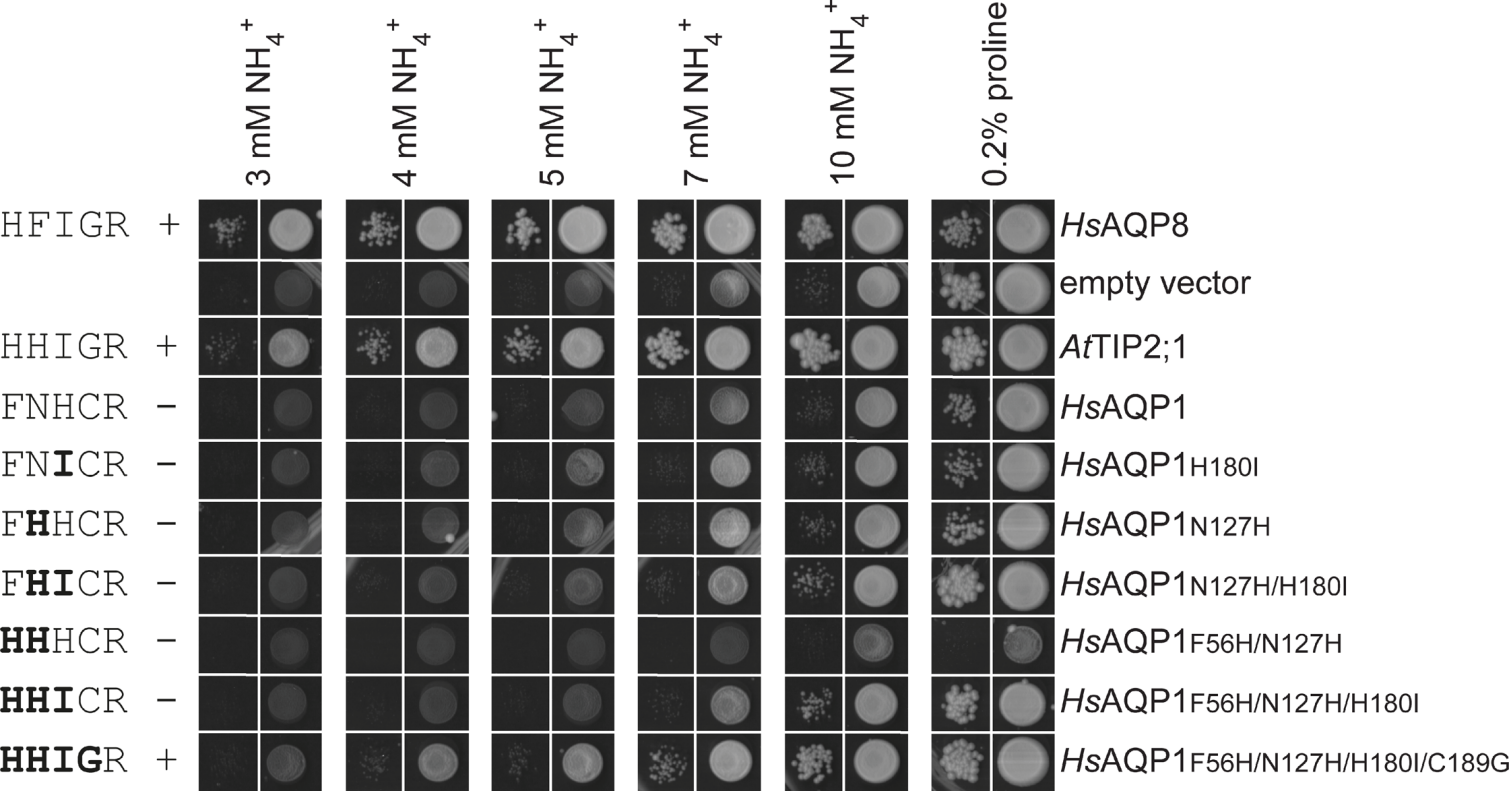
Growth complementation of ammonium uptake-defective yeast strain by mutants of *Hs*AQP1. The 31019b yeast strain (*Δmep1–3*) was transformed with the empty vector pYeDP60u or with pYeDP60u carrying cDNA encoding the positive controls *Hs*AQP8 or *At*TIP2;1, or *Hs*AQP1 or its mutants. The five amino acid residues of the extended selectivity filter in each construct are indicated in one letter code to the left in the order H2^P^, LC^P^, H5^P^, LE^P^, and HE^P^, showing substitutions in bold. Complementation and failure to complement are indicated by + and −, respectively. Transformants were spotted at an OD_600_ of 1 (right column) and 0.0001 (left column) on plates containing 0.2% proline or the indicated concentrations of ammonium as a sole nitrogen source and growth was recorded after 13 d at 28°C. Each panel showing growth at a specific concentration is compiled by individual pictures of each spot taken from a distinct growth condition. All single pictures were treated in the same manner.

The identification of determinants for substrate specificity based on functional knockout mutants is complicated by possible trivial explanations for loss of function, such as misfolding of the protein or failure to reach the plasma membrane. Therefore, rather than just trying to eradicate the ammonia specificity of *At*TIP2;1 by mutations, we chose to focus on gain-of-function mutants of the water-specific *Hs*AQP1 in an attempt to mimic the ammonia and water permeability of *At*TIP2;1. Hence, the four deviating residues in the extended selectivity filter of *Hs*AQP1 were mutated in a stepwise fashion to probe if a histidine at LC^P^, which may force the arginine at LE^P^ of *Hs*AQP1into an *At*TIP2;1-like position, was enough to achieve a matching substrate selectivity, or if additional substitutions were required. Unexpectedly, only the quadruple *Hs*AQP1 mutant (H2^P^-F56H, LC^P^-N127H, H5^P^-H180I, LE^P^-C189G) showed significant growth compared to the empty vector control in the yeast complementation assay for ammonia permeability (Fig 5). Stopped-flow experiments were conducted to quantitate ammonia and water permeability rates of the *Hs*AQP1 mutants and the controls. To compare relative specificities independently of differences in expression levels of the correctly folded protein in the yeast plasma membrane, the ratios of these rates were calculated (Table 2). Albeit the two rates represent alkalization and swelling rates in two different arbitrary units, their ratios offer an informative measure of the relative specificity in comparisons between mutated and wild type proteins. Whereas wild type *Hs*AQP1 only conducts water, *At*TIP2;1 showed a significant ammonia permeation rate, corresponding to approximately 3% of the water permeation rate in this strain. Interestingly, the same ratio was only obtained for the quadruple mutant of *Hs*AQP1, showing that these four substitutions are sufficient to reproduce an *At*TIP2;1-like specificity in *Hs*AQP1. Two other mutant forms of *Hs*AQP1, namely the H5^P^-H180I single mutant and the triple mutant (H2^P^-F56H, LC^P^-N127H, H5^P^-H180I), exhibited a higher specificity for ammonia than *At*TIP2;1 and similar absolute ammonia rates as the quadruple mutant, but surprisingly failed to complement the ammonia transport deletion strain in the growth assay. Insufficient water permeability of these mutants may explain these observations, implying that a dual permeability might be necessary for an efficient ammonia uptake in vivo. The impaired water permeability is likely to be a result of the higher hydrophobicity of isoleucine replacing the histidine at position H5^P^ of *Hs*AQP1. Apparently, water permeability is restored in the quadruple mutant by the LE^P^-C189G mutation. This is consistent with conservation of a small amino acid residue (alanine or glycine) at LE^P^ of TIPs. A small residue may allow the carbonyl oxygen in LE^P^—not hydrogen bonded to an amino acid at position LC^P^—to adopt the relaxed position and interact with waters in the selectivity filter more efficiently. In contrast to earlier mutational studies [4], our new structural insight has allowed us to rationally design and implant a TIP2-like substrate profile to a water-specific AQP.

**Table 2:** Transport rates for ammonia and water in yeast cells expressing *At*TIP2;1, *Hs*AQP1, or mutants of *Hs*AQP1 mimicking the selectivity filter of *At*TIP2;1.

Construct (SF position)	Ammonia × 10^−2^ (s^−1^)*	Water (s^−1^)*	Background corrected rates^†^ (s^−1^)		Ammonia/ water^‡^ × 10^−2^	Specificity^§^
			Ammonia × 10^−2^	Water		
Empty vector	6.7 ± 0.1	0.25 ± 0.01	0	0	NA	NA
*At*TIP2;1	39.1 ± 0.1	10.5 ± 0.1	32.4	10.2	3.2 ± 0.03	*At*TIP2;1-like
*Hs*AQP1 wt	6.3 ± 0.04	33.5 ± 0.2	0	33.2	0	Water
N127H (LC^P^)	4.7 ± 0.03	1.4 ± 0.001	0	1.13	0	Water
F56H (H2^P^) N127H (LC^P^)	6.1 ± 0.04	1.9 ± 0.01	0	1.62	0	Water
H180I (H5^P^)	11.5 ± 0.1	0.57 ± 0.0005	4.8	0.32	15 ± 1	Ammonia
N127H (LC^P^) H180I (H5^P^)	5.5 ± 0.03	0.31 ± 0.0004	0	0.05	NA	NA
F56H (H2^P^) N127H (LC^P^) H180I (H5^P^)	9.9 ± 0.1	0.33 ± 0.0005	3.2	0.07	44 ± 3	Ammonia
F56H (H2^P^) N127H (LC^P^) H180I (H5^P^) C189G (LE^P^)	12.4 ± 0.1	2.2 ± 0.02	5.7	1.91	3.0 ± 0.1	*At*TIP2;1-like

*Rates ± standard error of the fit calculated in GraphPad Prism.

^†^Rates for the strain with empty vector were subtracted for background correction. Negative rates were set to 0.

^‡^Background corrected rates were used to calculate the ratio as a measure of the substrate specificity. Ratios are presented ± propagated errors derived from given rates and their standard errors of the fit (see *).

^§^A lower ratio than *At*TIP2;1 is classified as water specificity, a higher as ammonia specificity, and a similar ratio as *At*TIP2;1-like.

NA, not applicable due to insignificant water and ammonia transport rates; wt, wild type.

**Fig 6:**
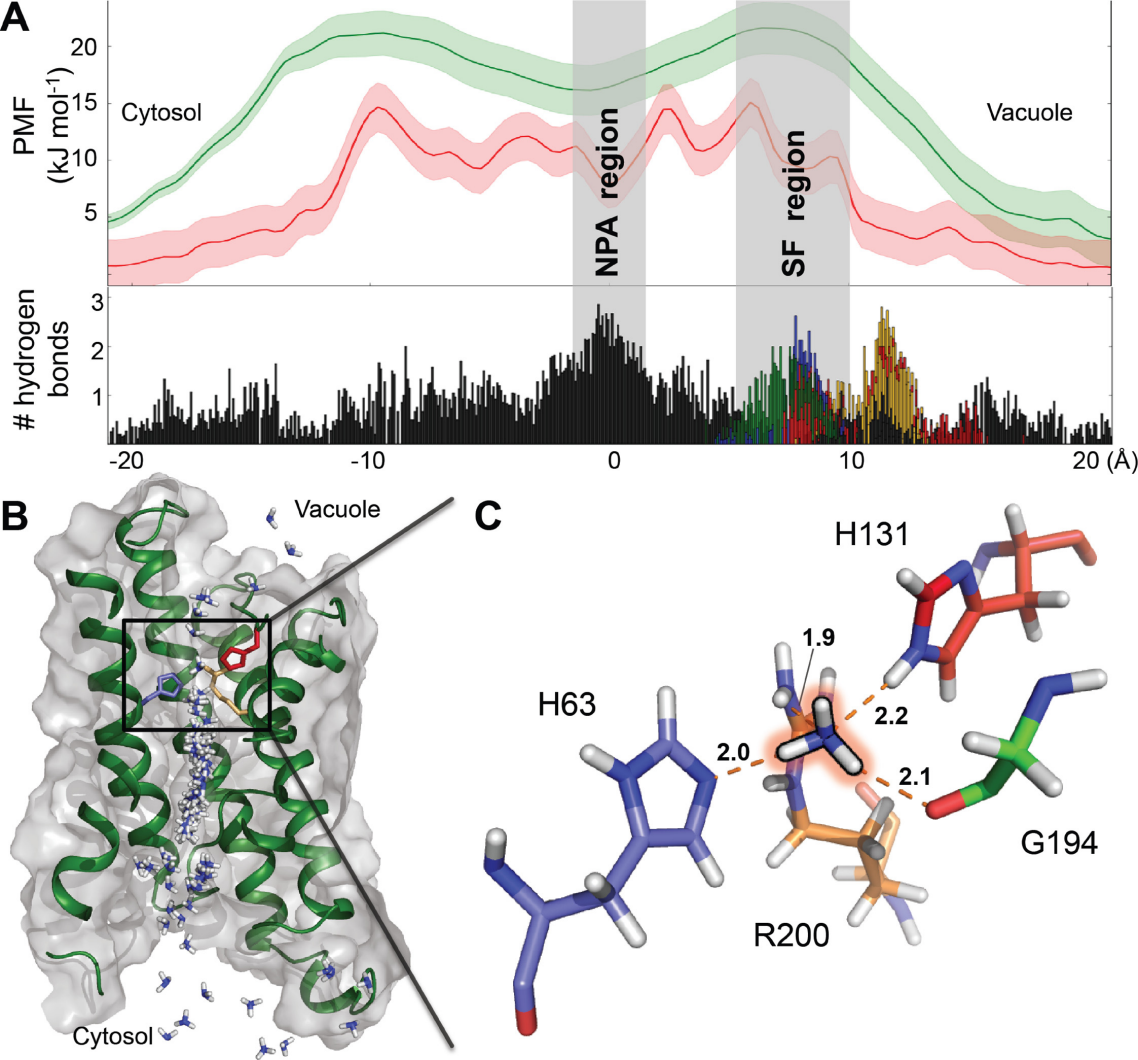
MD simulations of *At*TIP2;1. (A) The potential mean force (PMF) profiles for ammonia through *At*TIP2;1 (red) and through a model membrane containing 20% cholesterol (green). In the lower part of the panel, the number of hydrogen bonds between ammonia and *At*TIP2;1 are shown as function of position along the pore axis. Interactions with residues in the extended selectivity filter depicted in (C) are color-coded to resolve their contribution (H2^P^-His 63 (blue), LC^P^-His 131 (red), LE^P^-Gly 194 (green) and HE^P^-Arg 200 (brown)), and demonstrate hydrogen bonding to each of the four polar residues of the extended selectivity filter. (B) Snapshots of ammonia permeation. Cross section of *At*TIP2;1 shown as grey surface and green cartoon of the backbone. Side chains of selected amino acid residues in the selectivity filter are displayed as sticks and color coded as in (C). (C) Close-up of an ammonia molecule at the center, forming hydrogen bonds to four residues (H2^P^-His 63, LC^P^-His 131, LE^P^-Gly 194, and HE^P^-Arg 200) of the selectivity filter. The hydrogen bonds are indicated by orange dashes and distances are given in Å. Ile 185 at position H5^P^ of the selectivity filter, located in front of the visual plane, is not shown. The underlying data of panel A can be found in S1 Data.

Although the high resolution structure of *At*TIP2;1 allowed us to discriminate between nitrogen and carbon atoms in side chains of histidines (Fig 3B), it would not be possible to distinguish nitrogen of ammonia from oxygen of water in the pore of *At*TIP2;1 due to their similar electron density and expected low ammonia occupancy. To get a more detailed view of the substrate specificity in *At*TIP2;1, we therefore employed MD simulations. Water permeation was seen at high frequency (*p*_f_ ± SD) corresponding to approximately 25 ± 4 × 10^−14^ cm^3^ s^−1^ (S2 Fig), which is about four times as high as estimated for human AQP1. The high water permeation in *At*TIP2;1 is consistent with its low free energy for water (S3 Fig). Notably, spontaneous ammonia permeation events were observed in unbiased simulations with a length of 400 ns (S1 Movie) and verified by umbrella sampling simulations yielding a free energy barrier of approximately 15 kJ/mol (Fig 6A) in line with a high ammonia permeability. Further analysis shows that desolvation effects are compensated for by several hydrogen bonding residues at the selectivity filter (Fig 6A–6C), substantially lowering the energetic barrier in this region, where it peaks for the water-specific *Hs*AQP1 [25]. In contrast to a simple model membrane (S3 Fig), the tonoplast contains sterols [26], which increase the impermeability to polar molecules. Therefore, the ammonia permeability of *At*TIP2;1 is compared to a cholesterol containing model membrane with a free energy barrier for ammonia of 20 kJ/mol (Fig 6A). Due to these differences in energy barriers, the permeability of *At*TIP2;1 is an order of magnitude higher.

### Accumulation of Ammonium at the Surface

**Fig 7:**
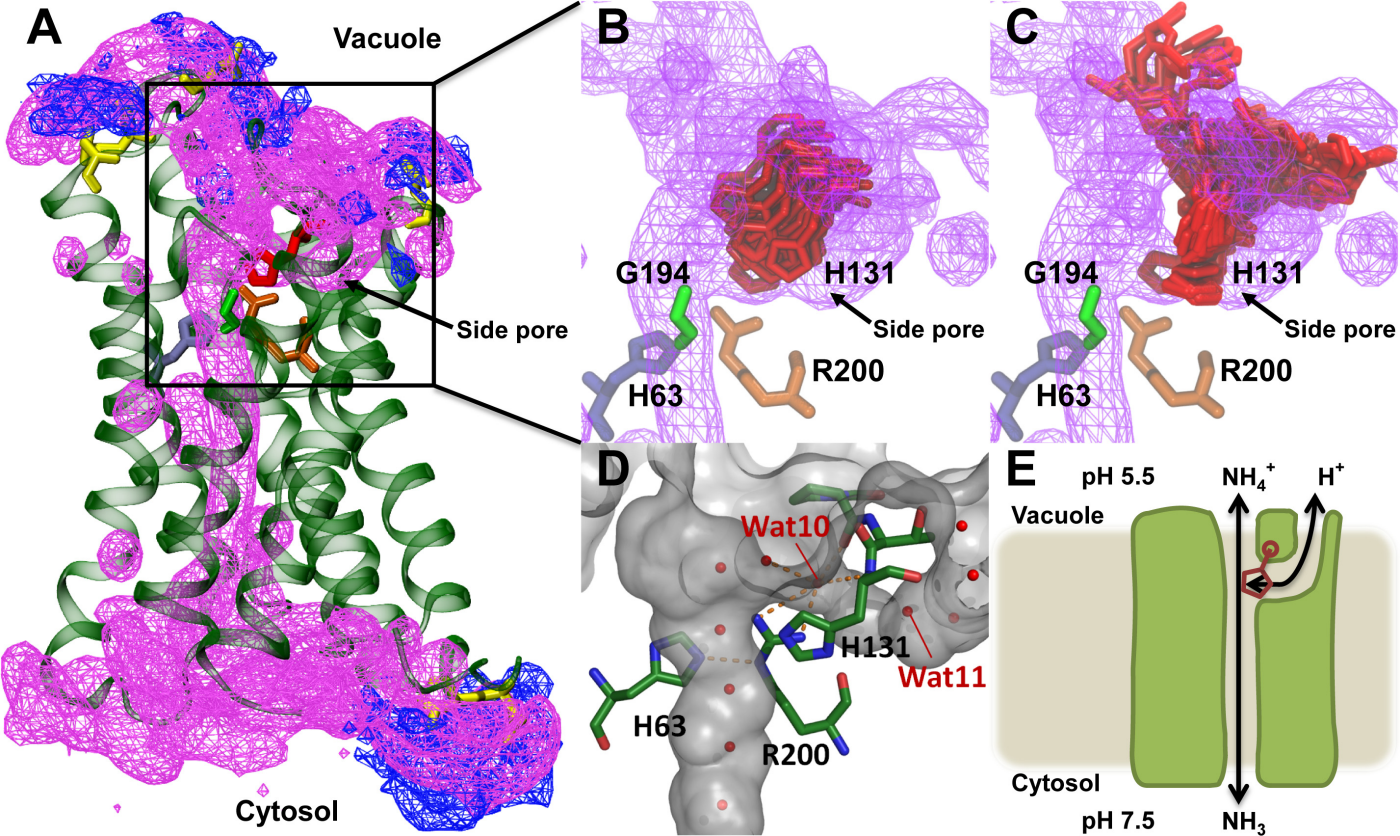
Ammonium accumulation and possible proton pathway. (A) MD simulations showing ammonium accumulation (blue mesh) at aspartate residues (yellow sticks) at vacuolar (top) and cytosolic (bottom) side of *At*TIP2;1. Water density (purple mesh) outlines the vertical main pore of the monomer and confirms existence of a water-filled side pore beneath loop C. Residues of the extended selectivity filter are depicted as sticks (H2^P^-His 63 (blue), LC^P^-His 131 (red), LE^P^-Gly 194 (green), and HE^P^-Arg 200 (brown)). (B) and (C) MD simulations demonstrating flexibility of His 131 at position LC^P^ being neutral (B) and positively charged (C). Color code as in (A). (D) Surface representation of the crystal structure depicting the water-filled side pore beneath loop C. Hydrogen bonds of water 10 (Wat10) as well as between Arg 200 at position HE^P^ in helix E and His 63 at position H2^P^ in helix 2 are indicated by dashed orange lines. (E) Tentative working model of ammonia-permeating *At*TIP2;1. Ammonium may contribute to ammonia permeation by accumulating on the vacuolar protein surface and by possibly having its protons shuttled back into the acidic vacuole by His 131 (red) at position LC^P^ in loop C via a water-filled side pore.

It is debated whether aquaammoniaporins are permeated by ammonium ions or not [27]. This has physiological relevance, since an effective exclusion of ammonium ions is necessary to acid-trap it in the vacuole. MD simulations containing ammonium ions showed no spontaneous permeation events, as expected due to electrostatic repulsion and desolvation effects in the pore. This brings to mind the ammonia transporter AmtB, which is generally considered to have a similar ammonium/ammonia selectivity, but where ammonia permeation has been proposed to be stimulated by ammonium recruitment to the noncytosolic vestibule [28]. We therefore investigated if ammonium ions accumulate on the vacuolar surface of *At*TIP2;1. A comparison of the electrostatics reveals that the vacuolar surface of *At*TIP2;1 is distinctly more negative than the corresponding surface of another plant AQP, the extracellular surface of the plasma membrane located water-specific *So*PIP2;1 that we established the structure of at 2.1 Å resolution [14] (S4 Fig). The negative vacuolar surface of *At*TIP2;1 is predominant at exposed acidic residues (Asp 48, Asp 52, Asp 210). Indeed, our MD simulations show a local enrichment of ammonium ions at these acidic residues (Fig 7A). Although the exact positions of acidic residues vary, a negative vacuolar surface constitutes a conserved feature among TIPs, which implies a generality of this finding.

### Conceivable Function of the Side Pore

Considering the low pH in some vacuoles and accumulation of ammonium on the vacuolar surface of *At*TIP2;1, the permeation efficiency would clearly benefit if ammonium contributes to channeling of ammonia. How then is ammonium deprotonated? The distal position of the second vacuolar loop (loop C) relative to Arg 200 at position HE^P^ in helix E hints at an explanation. In fact, structure and MD simulations concur that loop C leaves enough space for a continuous side pore reaching from the selectivity filter all the way to the vacuolar surface (Fig 7A–7D). In all previously reported AQP structures, the HE^P^-arginine is directly hydrogen bonded to the backbone carbonyl oxygen of the residue corresponding to Pro 129 of loop C. In *At*TIP2;1, this contact is instead mediated via a water molecule (Wat 10; Fig 7D) occupying a similar position as the arginine-binding carbonyl oxygen in other AQP structures. Interestingly, the peptide bond preceding the TIP2-specific histidine (His 131) at position LC^P^ in loop C retains an unusually large dihedral angle (19°) [29], and this contortion would be even larger with a deeper position of loop C. To explore if His 131 at position LC^P^ and the side pore could play a role in facilitating deprotonation of ammonium, we conducted further MD simulations. Ammonium deprotonation via LC^P^-His 131 through the side pore most likely requires a positional shift of this residue away from ammonia and towards a water molecule in the side pore (Wat 11) to get in hydrogen bond distance. The simulations indicate that the angle of the His 131 side chain (chi 1) remains as in the crystal structure when neutral (Fig 7B and S5 Fig), whereas in a protonated, i.e. positively charged state, an alternative orientation towards Wat 11 is indeed observed (Fig 7C, S5 Fig and S6 Fig). Furthermore, as simulations support the side pore as being continuously solvated, from His 131 to the vacuolar exit, it offers a proton wire for return of protons to the vacuolar environment, as ammonium is transferred to the pore as ammonia. These findings lead us to speculate about a possible TIP2-specific mechanism (Fig 7E) where histidine (His 131) at position LC^P^ in loop C shuttles protons from the main pore to the vacuolar surface via the side pore, using a Grotthuss mechanism, putatively enhancing the permeation rate of ammonia from the vacuole under nonequilibrium flux conditions.

## Discussion

The atomic structure of the water and ammonia permeable *At*TIP2;1 provides new insights into the substrate selectivity of AQPs. The structure reveals an extended selectivity filter, including a fifth amino acid residue at position LC^P^ in loop C that also may play a role in defining substrate profiles of the entire AQP superfamily. The importance of the extended selectivity filter is demonstrated by mutational studies in vivo and in vitro, showing gain-of-function of *At*TIP2;1 substrate selectivity in the water-specific human AQP1. MD simulations support ammonia conductance and a lack of ammonium permeability. As expected from the structure, ammonia interacts with LC^P^-His 131 and behaves similar to water in the pores of AQPs [18], reorienting in the NPA region at the N-termini of helix B and helix E due to their macro dipoles (S1 Movie). Based on structural analyses and simulations, we describe a selectivity filter that is highly permeable to ammonia due to its width and many potential polar contacts with the substrate and speculate on a mechanism in which ammonia permeation may be further increased by ammonium accumulation at the vacuolar protein surface, deprotonation through the TIP2-specific LC^P^-His 131, and proton transfer via a previously unidentified water-filled side pore. It should be stressed that there is only limited support from simulations for this speculation, and without confirmatory structures it is difficult to specifically target the side pore by mutations or to predict if it is a conserved feature of other TIPs and AQP8s. However, we find it most likely that the conserved arginine at position HE^P^ in helix E of other TIPs and AQP8s adopt the same conformation, as shown here for *At*TIP2;1, due to aromatic residues at position LC^P^ in loop C and hydrogen bonding residues at position H2^P^ in helix 2. The conservation patterns in the selectivity filter of AQP8s and separate subgroups of TIPs indicate that a functional differentiation has evolved among aquaammoniaporins. TIP2s and TIP4s of higher plants are similar to TIP6s found in primitive plants like mosses and are therefore likely to represent original functions and mechanistic features of TIPs. In contrast, TIP1s and TIP3s, which appear with the emergence of seed plants, as well as AQP8s in animals, lack the histidine at position LC^P^, which is proposed here to enhance deprotonation of ammonium. Such variations among aquaammoniaporins may relate to pH-dependent properties, which however remains to be investigated. In this context, it should be mentioned that both AQP8 and an AtTIP2;1 mutant lacking the histidine at position LC^P^ complemented ammonia permeability in the growth assay equally well or better than the wild type AtTIP2;1, demonstrating that a histidine at this position is not essential for efficient ammonia uptake under these conditions. The *At*TIP2;1 structure will facilitate modeling of other AQPs including human AQP8 and may therefore also help to elucidate the molecular basis of ammonia permeation in man.

From our results, it is clear that *At*TIP2;1 can enhance the ammonia permeability of membranes, but conditions linking an ammonia-related phenotype to TIPs have so far not been reported in plants [30]. Plants emit significant amounts of ammonia from their leaves, and ammonia generated by photorespiration further accentuate losses, implying a limited capacity of ammonia reassimiliation enzymes [31]. We expect that high ammonia permeability of the tonoplast and rapid acid-entrapment of ammonium in the vacuole is especially important under transient periods of photorespiration when it counteracts high levels of ammonia in the cytosol and thereby reduces losses over the plasma membrane, giving reassimilation pathways time to incorporate more of the generated ammonia. Hence, we propose that expression of ammonia-permeable mutant AQP isoforms in the plasma membrane, such as PIP2 mutants having a TIP2-like extended selectivity filter, combined with regulation of TIP expression can be used to vary the relative ammonia permeability of the plasma membrane and the tonoplast to explore effects on ammonia emission under these conditions. Control of ammonia emission by regulation of ammonia permeability in membranes could potentially open up a new way to improve the nitrogen use efficiency in plants.

## Materials and Methods

For details, see Supporting Information S1 Text.

*At*TIP2;1 with an N-terminal deca-His-tag was expressed in *Pichia pastoris* and purified as previously described [32]. Briefly, membranes were urea washed and solubilized with 10% n-octyl-β-D-glucoside (OG). The protein was purified by nickel affinity chromatography, employing a 100 mM imidazole wash, followed by size exclusion chromatography using an S200 column. Functionality was verified by stopped-flow analyses of proteoliposomes.

Hanging drop vapor-diffusion crystallization was performed at room temperature using a reservoir solution consisting of 50 mM magnesium/sodium acetate pH 5.0 and 28% (v/v) PEG 400 and crystals were flash-cooled in liquid nitrogen. Data were collected at 1 Å wavelength on the X06SA (PXI) beamline at the Swiss Light Source, Villigen, Switzerland. The structure was solved by molecular replacement and the model built manually. Refinement excluded 3% reflections, including twin-mates, and resulted in a twin fraction of 40.7%, reaching R_work_ and R_free_-values of 10.2% and 11.2%, respectively. Ramachandran outliers and residues in unfavored regions were manually inspected.

Mutant studies of *At*TIP2;1 and *Hs*AQP1 were executed using protoplasts and intact cells from *Saccharomyces cerevisiae*, as previously described [33].

The simulations were conducted with the GROMACS 4.5 software [34] using the CHARMM36 forcefield [35]. To study the properties of *At*TIP2;1, the protein was embedded in a 1-Palmitoyl-2-oleoylphosphatidylcholine (POPC) bilayer. Three unbiased 500 ns simulations were conducted to study the equilibrium behaviour of *At*TIP2;1, in the presence of water, ammonia or ammonium ions. Umbrella sampling was employed to calculate the PMF for permeation of water and ammonia [36,37].

## Supporting Information

S1 Data Numerical data used in preparation of Figs 2A–2D and 6A, S2, S3, S5 and S6 Figs. (XLSX)

S1 Fig Selectivity filter carbonyls in loop E cluster in two distinct spatial groups. (A) HE^P^-arginine (R200) of *At*TIP2;1 is shown for orientation. Carbonyls at LE^P^ of water-specific AQPs form group II (violet shading), and most of them are within hydrogen-bonding distance to two water molecules in their structures as illustrated by *So*PIP2;1 (PDB ID 1Z98; violet). Carbonyls of non-water-specific channels gather in a different location (group I; green shading). Among those are *At*TIP2;1 (green), glycerol transport facilitating, and uncharacterized proteins (*Af*AqpM, *Pf*AQP, *Ec*GlpF, *Mm*AqpM), but the water-specific *Hs*AQP4 also belongs to this group. Like all other members of this group, *Hs*AQP4 is lacking the LC^P^-asparagine (N153 in *So*PIP2;1, Fig 3D) that is conserved among the other water-specific proteins (blue shading, only asparagines residues are shown). Each carbonyl in group II can form a hydrogen bond to the carboxamide of this asparagine, if the carboxamide is oriented the right way. A certain flexibility is suggested by the special case of *Hs*AQP0, where different structures are available (1YMG and 2B6O shown) and the carbonyl is seen with both orientations. Apart from the glycerol facilitators, it appears that small residues like glycine and alanine in LE^P^ (Fig 3D) are required in group I, whereas slightly larger residues like cysteine or threonine can be accommodated in group II. Only backbone is shown in LE^P^. (B) Close up of *At*TIP2;1 (green) and *So*PIP2;1 (violet), showing hydrogen bonding of carbonyls at LE^P^ and water interacting with LC^P^-His 131. Side chain of LE^P^-Thr 219 is not shown. Main pore of *At*TIP2;1 analyzed by HOLE [38].
(TIF)

S2 Fig Osmotic permeability (*p*_f_) values calculated from MD simulations. *p*_f_ values were calculated separately for each monomer in seven 50-ns time windows. The contribution of the individual monomers to the *p*_f_ values of the tetramer are indicated by different colors and average values per monomer and standard deviation in each time window are indicated by the black line and error bars. The underlying data can be found in S1 Data.
(TIF)

S3 Fig PMF for water across the *At*TIP2;1 channel calculated from the equilibrium trajectory (black). The error bars are the standard deviation of the PMF over the four monomers of the protein. PMF profile for ammonia across a model membrane without cholesterol is shown in blue. The underlying data can be found in S1 Data.
(TIF)

S4 Fig Corresponding vacuum electrostatic maps of two plant AQPs. The noncytosolic surface of the tetramer of *So*PIP2;1 (left, facing the apoplast) and *At*TIP2;1 (right, facing the interior of the vacuole). Positive and negative electrostatic potentials calculated by PyMol [39] are marked by gradients of blue and red, respectively.
(TIF)

S5 Fig Dihedral populations of LC^P^-His 131 at three different protonated states. Chi1 angles in MD simulations with doubly protonated (positively charged; blue), Nδ protonated (neutral; yellow), and Nε protonated (neutral; red) His 131. The underlying data can be found in S1 Data.
(TIF)

S6 Fig Dihedral chi1 angle of the LC^P^-His 131 residue in the doubly protonated His 131 simulation summarized in S5 Fig. The data is shown for final ~350 ns of the 500 ns trajectory after the first observed transition. The His 131 residue spontaneously exchanges between its possible configurations in all the four monomers of the protein. The underlying data can be found in S1 Data.
(TIF)

S1 Movie MD simulation demonstrates spontaneous ammonia permeation. *At*TIP2;1 monomer is displayed in side view with LC^P^-His 131 of the selectivity filter as well as Asn 83 and Asn 197 in the NPA region shown as sticks. An ammonia molecule (sphere representation) enters the main pore from the cytosolic side of the protein. During permeation of the pore, ammonia reorients such that its free electron pair points towards the asparagines at the protein center. In the course of the video, which corresponds to 2.94 ns, the imidazole group of LC^P^-His 131 can be observed rotating by 180° and back again.
(MP4)

S1 Table Protein structures used in Fig 3A. (PDF)

S2 Table Logos and list of accession numbers for protein sequences used to generate Fig 3D. (PDF)

S3 Table List of primers used to generate wild type and mutated AQP constructs. (PDF)

S1 Text Supporting Materials and Methods. (DOCX)

## Abbreviations

AQP: aquaporin
MD: molecular dynamics
NPA: Asn-Pro-Ala
OG: n-octyl-β-D-glucoside
PIP: plasma membrane intrinsic protein
PMF: potential mean force
POPC: 1-Palmitoyl-2-oleoylphosphatidylcholine
TIP: Tonoplast Intrinsic Protein
