# Supplementary figures and images for: Crystal Structure of an Ammonia-Permeable Aquaporin
Source: PLoS Biol. 2016 Mar 30;14(3):e1002411. doi: 10.1371/journal.pbio.1002411 (PMC4814140; doi:10.1371/journal.pbio.1002411)

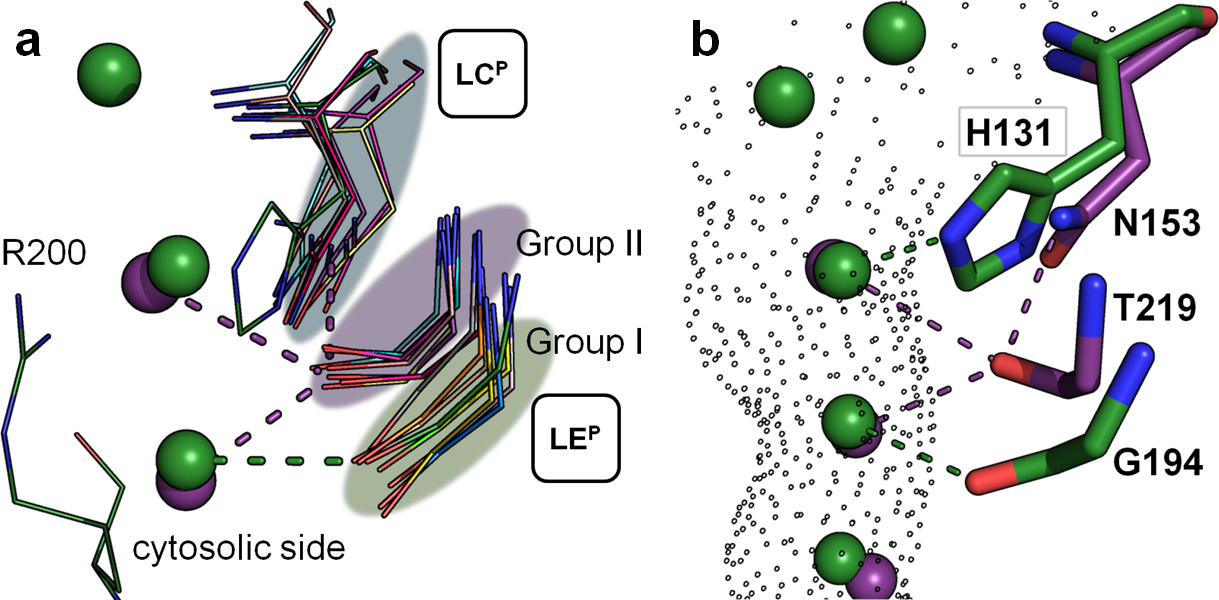

Supplement: S1 Fig — (A) HEP-arginine (R200) of AtTIP2;1 is shown for orientation. Carbonyls at LEP of water-specific AQPs form group II (violet shading), and most of them are within hydrogen-bonding distance to two water molecules in their structures as illustrated by SoPIP2;1 (PDB ID 1Z98; violet). Carbonyls of non-water-specific channels gather in a different location (group I; green shading). Among those are AtTIP2;1 (green), glycerol transport facilitating, and uncharacterized proteins (AfAqpM, PfAQP, EcGlpF, MmAqpM), but the water-specific HsAQP4 also belongs to this group. Like all other members of this group, HsAQP4 is lacking the LCP-asparagine (N153 in SoPIP2;1, Fig 3D) that is conserved among the other water-specific proteins (blue shading, only asparagines residues are shown). Each carbonyl in group II can form a hydrogen bond to the carboxamide of this asparagine, if the carboxamide is oriented the right way. A certain flexibility is suggested by the special case of HsAQP0, where different structures are available (1YMG and 2B6O shown) and the carbonyl is seen with both orientations. Apart from the glycerol facilitators, it appears that small residues like glycine and alanine in LEP (Fig 3D) are required in group I, whereas slightly larger residues like cysteine or threonine can be accommodated in group II. Only backbone is shown in LEP. (B) Close up of AtTIP2;1 (green) and SoPIP2;1 (violet), showing hydrogen bonding of carbonyls at LEP and water interacting with LCP-His 131. Side chain of LEP-Thr 219 is not shown. Main pore of AtTIP2;1 analyzed by HOLE [38]. (TIF) [file pbio.1002411.s002.tif]

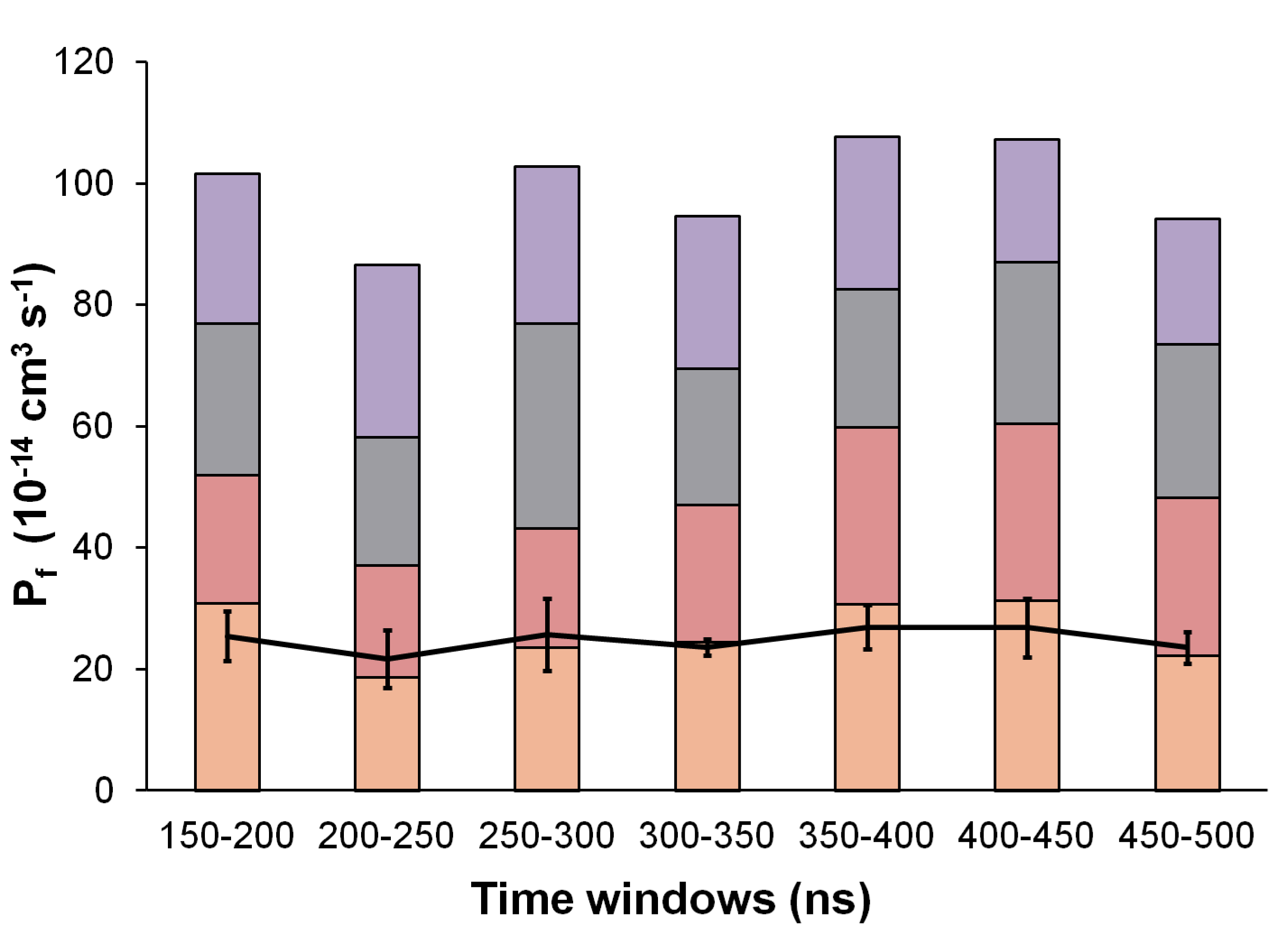

Supplement: S2 Fig — p f values were calculated separately for each monomer in seven 50-ns time windows. The contribution of the individual monomers to the p f values of the tetramer are indicated by different colors and average values per monomer and standard deviation in each time window are indicated by the black line and error bars. The underlying data can be found in S1 Data. (TIF) [file pbio.1002411.s003.tif]

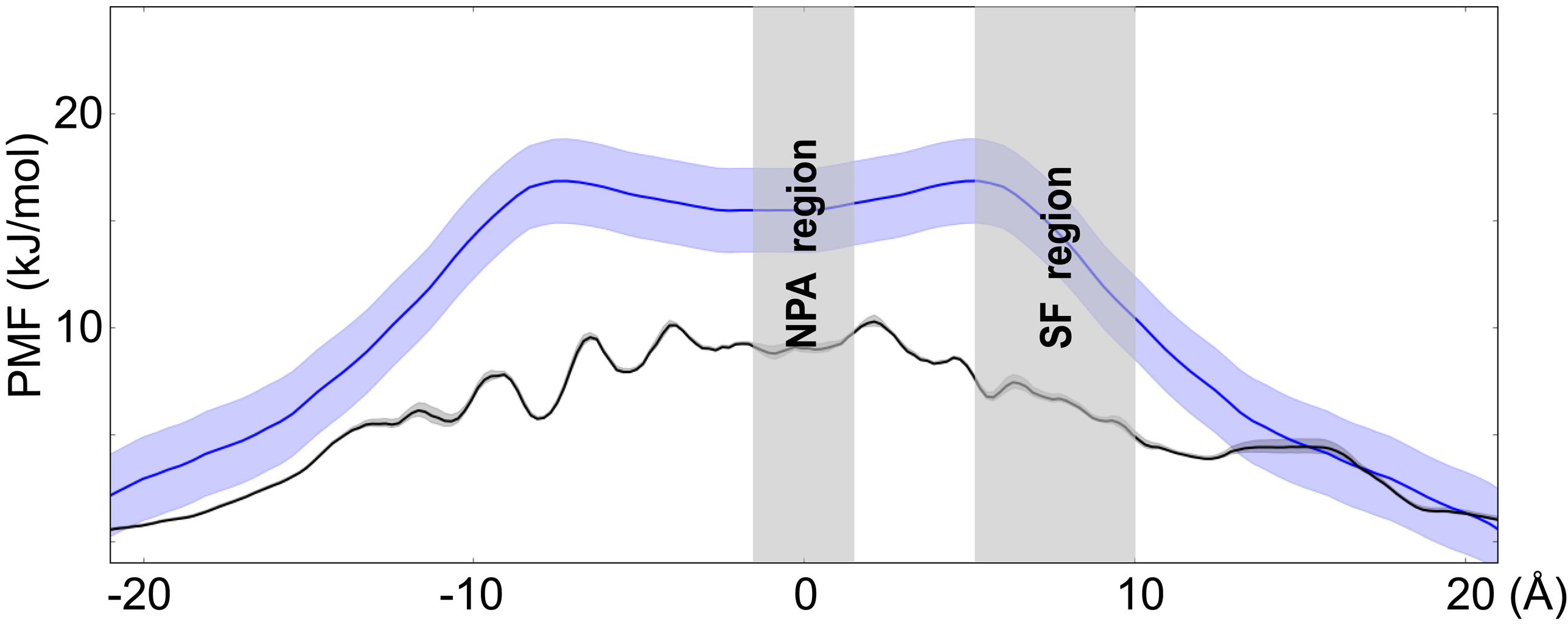

Supplement: S3 Fig — The error bars are the standard deviation of the PMF over the four monomers of the protein. PMF profile for ammonia across a model membrane without cholesterol is shown in blue. The underlying data can be found in S1 Data. (TIF) [file pbio.1002411.s004.tif]

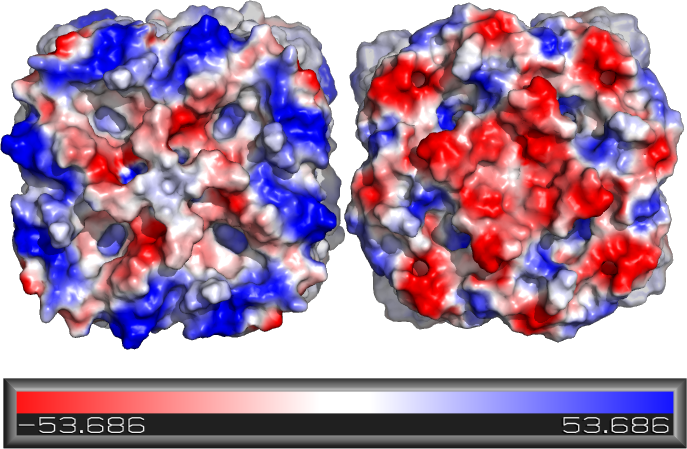

Supplement: S4 Fig — The noncytosolic surface of the tetramer of SoPIP2;1 (left, facing the apoplast) and AtTIP2;1 (right, facing the interior of the vacuole). Positive and negative electrostatic potentials calculated by PyMol [39] are marked by gradients of blue and red, respectively. (TIF) [file pbio.1002411.s005.tif]

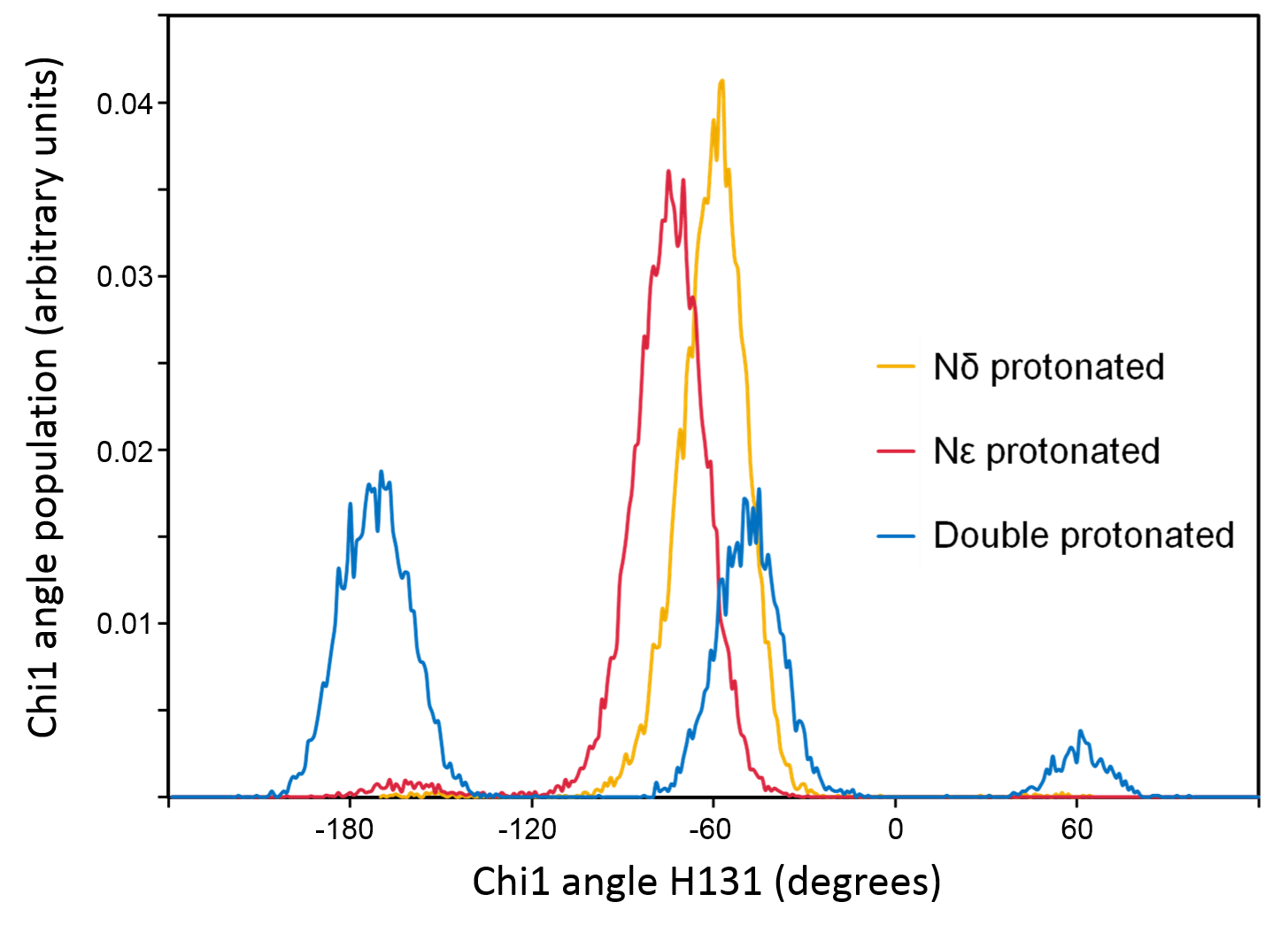

Supplement: S5 Fig — Chi1 angles in MD simulations with doubly protonated (positively charged; blue), Nδ protonated (neutral; yellow), and Nε protonated (neutral; red) His 131. The underlying data can be found in S1 Data. (TIF) [file pbio.1002411.s006.tif]

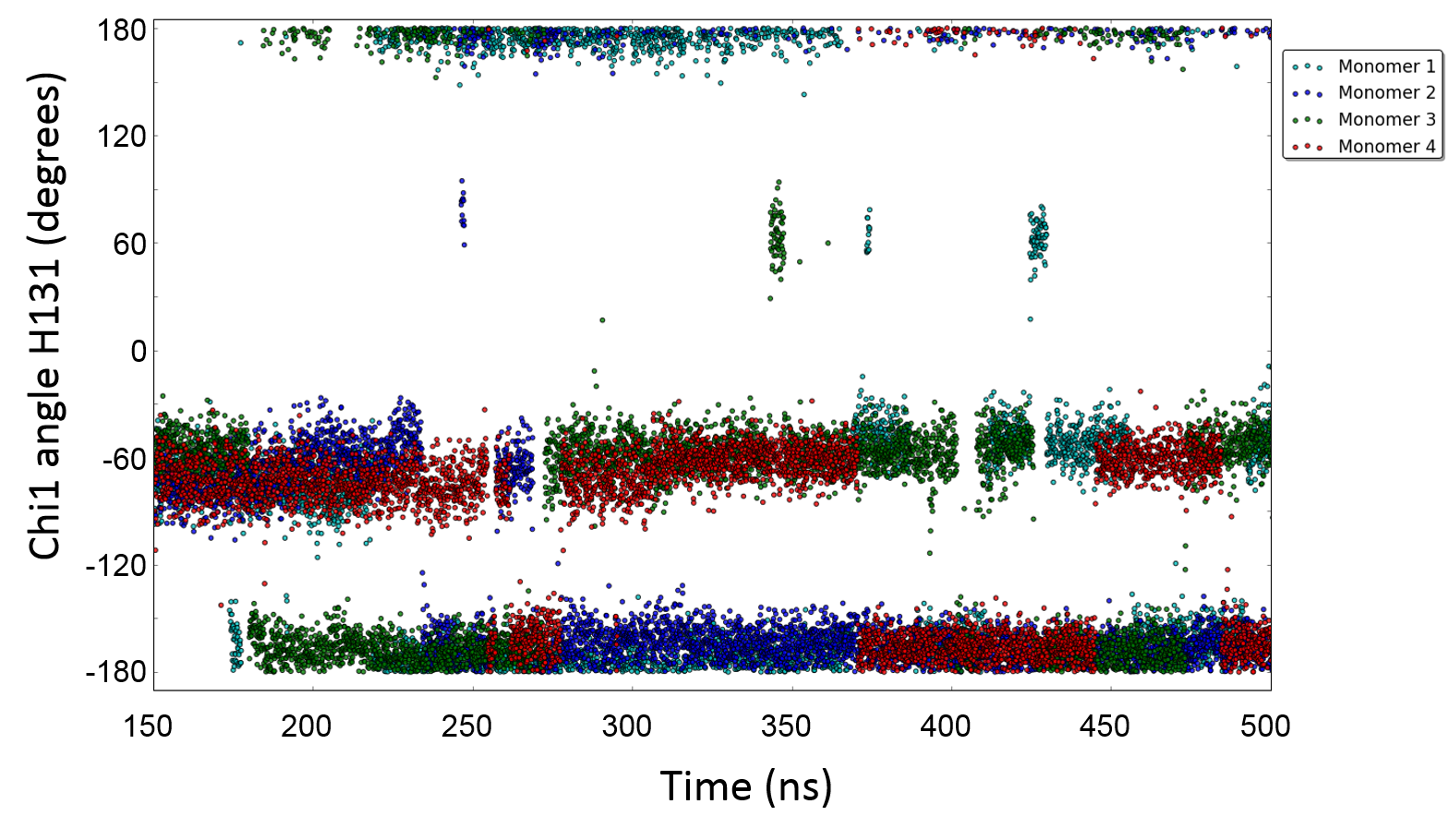

Supplement: S6 Fig — The data is shown for final ~350 ns of the 500 ns trajectory after the first observed transition. The His 131 residue spontaneously exchanges between its possible configurations in all the four monomers of the protein. The underlying data can be found in S1 Data. (TIF) [file pbio.1002411.s007.tif]
