# Supplementary material for: Crystal Structure of an Ammonia-Permeable Aquaporin
Source: PLoS Biol. 2016 Mar 30;14(3):e1002411. doi: 10.1371/journal.pbio.1002411 (PMC4814140; doi:10.1371/journal.pbio.1002411)
Supplement: S1 Table — (PDF) [file pbio.1002411.s009.pdf]

S1 Table: Protein structures used in Fig. 2A.

Protein name	PDB ID
*Ec*GlpF (with glycerol)	1FX8
*Hs*Aqp4	3GD8
*So*PIP2;1 (closed)	1Z98
*At*AqpZ 2*	3LLQ
*Pp*Aqy1*	2W2E
*Hs*Aqp5*	3D9S
*Ec*AqpZ*	1RC2
*Bt*Aqp1*	1J4N

*Used for calculation of average pore diameter
