# Supplementary material for: Crystal Structure of an Ammonia-Permeable Aquaporin
Source: PLoS Biol. 2016 Mar 30;14(3):e1002411. doi: 10.1371/journal.pbio.1002411 (PMC4814140; doi:10.1371/journal.pbio.1002411)
Supplement: S2 Table — (PDF) [file pbio.1002411.s010.pdf]

S2 Table: Logos and list of accession numbers for protein sequences used to generate Fig. 2D.

AQP*	Logo†	Reference/accession numbers
TIP2s	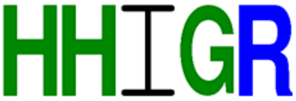	Anderberg et al. (32)
TIP4s	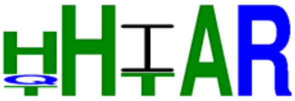	Anderberg et al. (32)
TIP6s	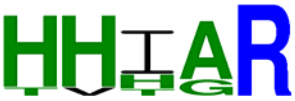	Anderberg et al. (32)
TIP3s	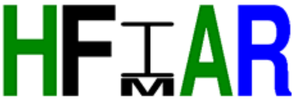	Anderberg et al. (32)
TIP1s	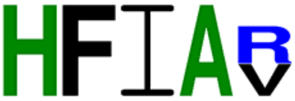	Anderberg et al. (32)
AQP8s	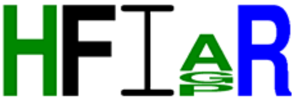	*Homo sapiens* NP_001160.2, *Rattus norvegicus* NP_062031.1, *Gallus gallus* XP_414866.2, *Xenopus tropicalis* NP_001107728.1, *Danio rerio* NP_001004661.1
PIPs	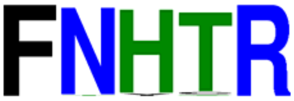	Anderberg et al. (32)
AQP1,2,5,6s	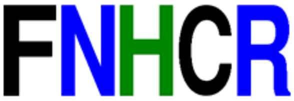	*Homo sapiens* AAH22486.1, NP_000477.1, NP_001642.1, AAB41566.1, *Rattus norvegicus* NP_036910.1, NP_037041.2, NP_036911.1, NP_071517.1, *Gallus gallus* NP_001034542.1, XP_428855.4, XP_001231781.2, *Xenopus tropicalis* NP_001005829.1, NP_001015749.1, XP_002935789.1, *Anguilla japonica* BAC82109.1
AQP0s	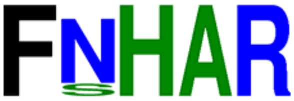	*Homo sapiens* NP_036196.1, *Bos taurus* NP_776362.1, *Ovis aries* NP_001153230.1, *Gallus gallus* NP_989597.1, *Xenopus laevis* NP_001088304.1, *Danio rerio* NP_001018356.1
AQP4s	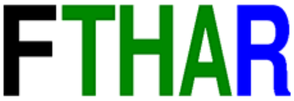	*Homo sapiens* NP_001641.1, *Rattus norvegicus* NP_036957.1, *Ovis aries* NP_001009279.1, *Gallus gallus* NP_001004765.1, *Anolis carolinensis* XP_003219692.1, *Strongylocentrotus purpuratus* XP_799266.2

*Subgroup or subfamily of AQPs. †Logos of residues at $H2^P$, LC^P, $H5^P$, LE^P and HE^P created by WebLogo3.3 (33). Character height is proportional to information content (bits) and colors reflect hydrophobicity. Only amino acid residues conserved in at least 50% of the sequences in the group are shown in Fig. 2D.
