# Supplementary material for: Crystal Structure of an Ammonia-Permeable Aquaporin
Source: PLoS Biol. 2016 Mar 30;14(3):e1002411. doi: 10.1371/journal.pbio.1002411 (PMC4814140; doi:10.1371/journal.pbio.1002411)
Supplement: S3 Table — (PDF) [file pbio.1002411.s011.pdf]

S3 Table: List of primers used to generate wild type and mutated aquaporin constructs.

**Name**	**Sequence**
**AtTIP2;1_Fw**	GGATTAAUAATGGCTGGAGTTGCCTTTGG
**AtTIP2;1_Rv**	GGGTTAAUTTAGAAATCAGCAGAAGCAAG
**HsAQP1_Fw**	GGATTAAUAATGGCCAGCGAGTTCAAGAAG
**HsAQP1_Rv**	GGGTTAAUCTATTTGGGCTTCATCTCCACC
**HsAQP1H180I_Fw**	ATTCTCCTGGCUATTGACTACACTGGCTGT
**HsAQP1H180I_Rv**	AGCCAGGAGAAUTCCAAGGGCTACAGAG
**HsAQP1N127H_Fw**	ACTCGCTTGGUCGCCATGACCTGGCTGATGG
**HsAQP1N127H_Rv**	ACCAAGCGAGUTCCCAGTCAGGGAGGAGG
**HsAQP1H180I/C189G_Fw**	ATTCTCCTGGCUATTGACTACACTGGCGGT
**HsAQP1F56H_Fw**	AGGTGTCGCUGGCCCATGGGCTGAGCATCGCCACGCTG
**HsAQP1F56H_Rv**	AGCGACACCUTCACGTTGTCCTGGACCGCC
**AtTIP2;1H63F_Fw**	ATCGCGGTTUGTTTCGGTTTTGCTCTCTTCGTGGC
**AtTIP2;1H63F_Rv**	AAACCGCGAUGGCCACTAGTCCCGGTGTATC
**AtTIP2;1H113N_Fw**	ACCAACAGCGUTGCGGCTGGACTAG
**AtTIP2;1H113N_Rv**	ACGCTGTTGGUTGGAACCGCCAATCCACCG
**AtTIP2;1I185H_Fw**	ACCTCGCCGCUGGTCCATTCTCCGGTGGATC
**AtTIP2;1I185H_RV**	AGCGGCGAGGUGGTTGGCACCAACGATAAGAC
**AtTIP2;1G194C_Fw**	ATTCTCCGGUTGTTCCATGAACCCAGCACGTTC
**AtTIP2;1G194C_Rv**	ACCGGAGAAUGGACCGGCGGCGAGGATG
**AtTIP2;1I185H/G194C_Fw**	ACCTCGCCGCUGGTCCATTCTCCGGTTGTTCCATGAAC
