# Supplementary material for: Crystal Structure of an Ammonia-Permeable Aquaporin
Source: PLoS Biol. 2016 Mar 30;14(3):e1002411. doi: 10.1371/journal.pbio.1002411 (PMC4814140; doi:10.1371/journal.pbio.1002411)
Supplement: S1 Text — (DOCX) [file pbio.1002411.s012.docx]

**S1 Text.** Supporting Materials and Methods

**Protein expression and purification**

A generic vector was constructed by modification of pPICZB (Invitrogen), introducing a deca-His-tag followed by a spacer 3’ of the MCS (FOR_TEV). The *At*TIP2;1 sequence with an N-terminal TEV-site was cloned into FOR_TEV and transformed into *Pichia pastoris*. A culture of a high-expressing clone was grown in a 3 L bioreactor (HANNA, Belach Bioteknik) and induced by continuous feed of methanol. Cells were harvested after 2 days induction at an OD_600_ of 328 and the overexpressed protein was purified as previously described ([1](#_ENREF_1)). Briefly, bead-beater-extracted membranes were washed with 4 M urea ([2](#_ENREF_2)) and solubilized at room temperature in Buffer A (20 mM Hepes pH 7.8, 50 mM NaCl) supplied with 10% n-octyl-β-D-glucoside (OG), 2 mM β-mercapto-ethanol and 1 mM PMSF. *At*TIP2;1 was purified in Buffer A supported by either 0.6% n-nonyl-β-D-glucoside (NG) for mercury inhibition assays or 0.8% OG for other liposome assays and crystallization. Solubilized membranes were bound to Ni-NTA beads (Qiagen) for at least two hours at 4°C. Ni-affinity chromatography was done on column maintaining reducing conditions of 4 mM β-mercapto-ethanol. Bound proteins were washed with 100 mM imidazole prior to elution. Eluted proteins were immediately separated further in Buffer A containing 2 mM dithiothreitol (DTT) by size exclusion chromatography on a S200 (GE Healthcare) at 4°C. The tetrameric fraction was concentrated to 13 – 17 mg protein/mL and stored at 4°C.

**Crystallization and structure determination**

Vapor-diffusion crystallization was performed at room temperature with 1 + 1 µL hanging drops. Several initial crystal hits were found within 3 – 7 days in MemGold (Molecular Dimensions) and PEGRx2 screen (Hampton Research). Initial diffraction was seen at beamlines I911-2 and -3 at Max-Lab, Lund, Sweden. Data for the presented structure was collected at 1 Å wavelength on the X06SA (PXI) beamline at the Swiss Light Source, Villigen, Switzerland on a crystal grown over a reservoir solution of 50 mM magnesium/sodium acetate pH 5.0 and 28% (v/v) PEG 400. Data was integrated with XDS ([3](#_ENREF_3)) and molecular replacement was based on the structure of *Hs*AQP4 PDB ID code 3GD8). Refinement was done with Refmac5 initially using detwinned data (detwin) and manually build with Coot([4](#_ENREF_4)) included in CCP4 package ([5](#_ENREF_5), [6](#_ENREF_6)). For further refinement 3% reflections (including twin-mates) were excluded from original twinned data for R_free_. Final refinement included hydrogens and resulted in a twin fraction of 40.7%. Structures were analyzed and pictures created in PyMOL ([7](#_ENREF_7)). Rampage ([8](#_ENREF_8)) locates 233 amino acid residues (98.7%) in the favored and 3 (1.3%) in the allowed region and crystallographic statistics calculated by MolProbity ([9](#_ENREF_9)) are found in Table 1. Pore diameters of selected AQPs (Table S1) were analyzed with the program HOLE ([10](#_ENREF_10)) excluding waters and non-proteogenic molecules.

**Functional assays of purified protein**

For kinetic assays, liposomes with and without protein were made by dialysis. For water transport assays at different pH, 66 µg protein and 2 mg *E. coli* POLAR lipids (Avanti), lipid-to-protein-ratio 30 (LPR 30), were dialyzed per mL Tris-buffer (20 mM pH 8.0, 100 mM NaCl, 2 mM DTT). Likewise for mercury inhibition, His-trap purified and desalted (PD-10, GE Healthcare) protein was reconstituted in liposomes at LPR 50. Vesicles for ammonia transport (LPR 16) were reconstituted in MOPS buffer (5 mM pH 5.6, 100 mM NaCl, 2 mM DTT) containing 0.5 mM carboxy-fluorescein. To determine water permeability, extruded vesicles (9 times by 200 nm filter) were diluted 10 times in either Tris-buffer (20 mM pH 8.0, 100 mM NaCl) or MOPS buffer (50 mM pH 5.0, 100 mM NaCl) resulting in pH 5.4. The diluted proteoliposomes were subjected to a 100 mM sorbitol gradient in a stopped-flow device (SF-61DX2, TgK Scientific Limited), and out-flow of water was followed by observing 90°-scattering at 500 nm. To inhibit protein-facilitated transport, proteoliposomes at pH 8.0 (LPR 50) were preincubated 30 min with 1 mM HgCl_2_. A single exponential decay function was fitted to an average of 7–15 curves by Kinetic studio (TgK Scientific Limited). Vesicles to measure ammonia transport were pelleted by centrifuging 1 h at 145,000×g and 4°C, washed with 10 mL buffer without carboxy-fluorescein and centrifuged again immediately before use. The pellet was resuspended with MOPS buffer to a final lipid concentration of 0.2 mg/mL and filtered with a 200 nm syringe filter (Sarstedt). In stopped-flow experiments, NaCl was partially exchanged for NH_4_Cl, and a change in fluorescence was recorded at an excitation of 490 nm using a cut-off filter of <495 nm. When NH_3_, which constitutes approximately 0.022% of the total NH_4_^+^/NH_3_ pool at pH 5.6, permeates the membrane the weakly buffered solution in the liposomes is alkalized and the quantum yield of carboxy-fluorescein increases. The single exponential rate constant of a decay function was fitted to ca. 10 curves between 3.8 ms (delay time) and 50 ms using GraphpadPrism ([11](#_ENREF_11)).

### Generation of constructs for mutational studies

The PCR products for mutated and non-mutated aquaporin constructs were directionally sub-cloned, using a uracil excision-based improved high-throughput USER cloning technique ([12](#_ENREF_12)), into the USER-compatible yeast expression vector pYeDP60u ([13](#_ENREF_13)). Point mutations were introduced into *Hs*AQP1 by PCR using the corresponding mutation primers (Table S3). The different PCR products were assembled into pYeDP60u using the efficient method for simultaneous fusion and cloning of multiple PCR products ([14](#_ENREF_14)). All constructs were verified by DNA sequencing.

### Yeast strain and growth assay

The *Saccharomyces cerevisiae* yeast strain 31019b ([15](#_ENREF_15)) was transformed with pYeDP60u or pYeDP60u containing cDNA encoding the respective aquaporin isoforms or their mutants. Transformants were spotted on synthetic medium containing 2% galactose, 50 mM succinic acid / Tris base, pH 5.5, 0.7% yeast nitrogen base without amino acids and ammonium (Difco) supplemented with 0.2% proline or different concentrations of (NH_4_)_2_SO_4_ as a sole nitrogen source. After 9 to 13 days of incubation at 28°C, differences in growth and survival in the complementation assays were recorded. The yeast growth assay was repeated in four independent experiments with consistent results.

**Permeability of yeast cells and protoplasts**

Overexpression in yeast and preparation of intact cells and protoplast for stopped-flow experiments was performed as previously described ([16](#_ENREF_16)). Briefly, *S. cerevisiae* strains containing vectors to express the different constructs were grown in 5 mL liquid SD-ura medium overnight at 30°C. 200 µL of each of these precultures were used to inoculate 25 mL SGal-ura medium and incubated for 48 h at 30°C. For preparation of protoplasts, 10 mL of each culture was pelleted gently (500×g, 5 min) and cells were washed with 4 mL equilibration buffer (50 mM potassium phosphate pH 7.2, 40 mM β-mercapto-ethanol). Cell walls were degraded for 30 min at 30°C in digestion buffer (equilibration buffer complemented with 2.4 M sorbitol, 50 mg/mL bovine serum albumin, and 0.5 mg/mL Zymolyase 20T). Protoplast were washed with 4 mL buffer 1A (10 mM Tris pH 8.0, 50 mM NaCl, 5 mM CaCl_2_, 1.8 M sorbitol), resuspended in 1 mL buffer 1A and stored on ice until used. To determine water permeability, protoplasts were subjected to a 300 mM hypo-osmolar sorbitol gradient and water influx was followed by recording scattering (see above). A single exponential decay function was fitted to 12 – 16 normalized curves by GraphPad Prism ([11](#_ENREF_11)).

To prepare samples for ammonia measurements, 10 mL intact cells per construct were washed in loading buffer (50 mM Hepes pH 7.0, 5 mM 2-deoxy-glucose) and incubated in 50 µM fluorescein diacetate for 20 to 30 min at 30°C. Fluorescein loaded cells were washed with buffer 2A (5 mM Tris pH 8.0, 50 mM NaCl) and stored at 4°C in the dark until use. Cells were washed again prior to the stopped-flow experiment, if not used immediately after dye load. Fluorescein loaded intact yeast cells were rapidly mixed with equal volumes of buffer 2B (as buffer 2A, but NaCl exchanged for 50 mM NH_4_Cl) creating a 1.3 mM inward ammonia gradient. Increased fluorescence (excitation wavelength 490 nm) upon alkalization was detected at 90° angle behind a long-pass filter with a cut-on wavelength of 530 nm. Between 2 and 14 curves were normalized per sample to fit an exponential function in GraphPad Prism.

**MD simulations**

The simulations were conducted with GROMACS 4.5 ([17](#_ENREF_17)). The protein was embedded in a 1-Palmitoyl-2-oleoylphosphatidylcholine (POPC) bilayer of 304 molecules using g_membed ([18](#_ENREF_18)). The system was simulated in the presence of 28,566 explicit molecules of TIP3P water ([19](#_ENREF_19)) and an ion concentration of 0.15 M KCl. The temperature was kept at 300 K using a Velocity rescale thermostat ([20](#_ENREF_20)) and the pressure was similarly maintained at 1 atmosphere using a Berendsen barostat ([21](#_ENREF_21)). The CHARMM36 forcefield was used for all simulations ([22](#_ENREF_22)). The electrostatic interactions in the system were treated explicitly with a PME method using a real space cut-off of 1.2 nm ([23](#_ENREF_23)). The Lennard-Jones interactions were treated with a switch function with the switch at a distance of 0.8 nm and a cut-off at 1 nm. The parameters used for simulating the ammonia were obtained from the charmm small molecules library ([24](#_ENREF_24)). The ammonium parameters were obtained by modifying the methylammonium ion parameters ([24](#_ENREF_24)). The lipid bilayers were created with the CHARMM-GUI web application ([25](#_ENREF_25)).

Three unbiased 500 ns simulations were run, studying the equilibrium behavior of the channel. The first of these was performed in the presence of water only to calculate the osmotic permeability (*p*_f_). This was done using the collective diffusion method ([26](#_ENREF_26), [27](#_ENREF_27)). To calculate the averages for the *p*_f_, the last 350 ns of the simulation were taken and divided into seven sections of 50 ns each. The *p*_f_ was separately computed for each monomer and the average over the four monomers per section was used as the measure of the *p*_f_ and the standard deviation was used to estimate the statistical error. In conjunction with this first unbiased simulation, two additional simulations were conducted for 300 ns to study the effect of different protonation states on the chi1 angle populations of His 131. From these simulations the first 100 ns were discarded to account for the equilibration and the last 200 ns were used for analysis.

The second and the third unbiased simulations were carried out in the presence of 100 molecules of ammonia and ammonium ions, respectively. The molecules were equally distributed into the two compartments formed by the bilayer. In these simulations the first 100 ns were discarded to allow for equilibration. The densities for the ammonium ions and water were plotted with the volmap tool from VMD ([28](#_ENREF_28)).

To calculate the Potential of Mean Force (PMF) for the permeation of ammonia, the g_wham tool from GROMACS 4.5 was used ([29](#_ENREF_29)) in the same manner as used by Hub *et al.* ([30](#_ENREF_30)). In order to ensure that the comparison between the PMFs presented is meaningful we use the cylindrical restraints as used by Hub *et al*. ([31](#_ENREF_31)). In this framework we introduce a flat bottom potential around the ammonia molecule, which restricts the target of umbrella sampling within a radial boundary of radius 2 nm. Within its boundary it experiences zero potential. Outside this boundary it experiences a harmonic potential with a force constant of 1,000 kJ mol^-1^ nm^-2^. This precaution ensures that the entropic effects of entering the channel pore are meaningfully accounted for when comparing different PMF profiles for *At*TIP2;1. We do not symmetrize the potentials. The model membrane consisted of equal amounts of POPC and 1,2-dipalmitoyl-glycero-3-phosphocholine (DPPC) lipids, with or without 20% cholesterol. The channel axis along the z coordinate was used for sampling. It was divided into 280 windows of 0.25 Å each to yield a length of 7 nm centered at the NPA motifs. Each window was simulated for 1.5 ns, 500 ps of which were discarded to allow for equilibration. A force constant of 1,000 kJ mol^-1^nm^-2^ was used to restrain the ammonia molecule in the window. The errors were calculated with the 50 iterations of the bootstrap algorithm as implemented in the g_wham tool ([29](#_ENREF_29)).

Images in Fig. 4A‒C and Movie S1 were created with the VMD program ([28](#_ENREF_28)). Movie S1 shows 147 consecutive frames, which are 20 ps apart from each other. For clarity, the trajectory of cartoon representation is smoothened with a window size of 5. The trajectories of amino acid residues highlighted as sticks are averaged over 2 frames (window size 1), while the ammonia molecule is depicted frame wise.

**References**

1. Karlsson M*, et al.* (2003) Reconstitution of water channel function of an aquaporin overexpressed and purified from Pichia pastoris. *FEBS Letters* 537(1-3):68-72.

2. Hasler L*, et al.* (1998) Purified lens major intrinsic protein (MIP) forms highly ordered tetragonal two-dimensional arrays by reconstitution. *Journal of Molecular Biology* 279(4):855-864.

3. Kabsch W (2010) Xds. *Acta Crystallogr D Biol Crystallogr* 66(Pt 2):125-132.

4. Emsley P, Lohkamp B, Scott WG, & Cowtan K (2010) Features and development of Coot. *Acta Crystallographica Section D* 66(4):486-501.

5. Corporate A (1994) The CCP4 suite: programs for protein crystallography. *Acta Crystallogr D Biol Crystallogr* 50(Pt-5):760-763.

6. Potterton E, Briggs P, Turkenburg M, & Dodson E (2003) A graphical user interface to the CCP4 program suite. *Acta Crystallographica Section D* 59(7):1131-1137.

7. Schrodinger, LLC, The PyMOL Molecular Graphics System, Version 1.5.0.4 (2010)).

8. Lovell SC*, et al.* (2003) Structure validation by Cα geometry: ϕ,ψ and Cβ deviation. *Proteins: Structure, Function, and Bioinformatics* 50(3):437-450.

9. Chen VB*, et al.* (2010) MolProbity: all-atom structure validation for macromolecular crystallography. *Acta Crystallogr D Biol Crystallogr* 66(Pt 1):12-21.

10. Smart OS, Goodfellow JM, & Wallace BA (1993) The pore dimensions of gramicidin A. *Biophys J* 65(6):2455-2460.

11. GraphPad Software SDCU, [www.graphpad.com](http://www.graphpad.com) (2007) GraphPad Prism version 5.01 for Windows.

12. Nour-Eldin HH, Hansen BG, Norholm MH, Jensen JK, & Halkier BA (2006) Advancing uracil-excision based cloning towards an ideal technique for cloning PCR fragments. *Nucleic Acids Res* 34(18):e122.

13. Hamann T & Moller BL (2007) Improved cloning and expression of cytochrome P450s and cytochrome P450 reductase in yeast. *Protein Expr Purif* 56(1):121-127.

14. Geu-Flores F, Nour-Eldin HH, Nielsen MT, & Halkier BA (2007) USER fusion: a rapid and efficient method for simultaneous fusion and cloning of multiple PCR products. *Nucleic Acids Res* 35(7):e55.

15. Marini AM, Soussi-Boudekou S, Vissers S, & Andre B (1997) A family of ammonium transporters in Saccharomyces cerevisiae. *Mol Cell Biol* 17(8):4282-4293.

16. Bertl A & Kaldenhoff R (2007) Function of a separate NH3-pore in Aquaporin TIP2;2 from wheat. *FEBS Letters* 581(28):5413-5417.

17. Pronk S*, et al.* (2013) GROMACS 4.5: a high-throughput and highly parallel open source molecular simulation toolkit. *Bioinformatics* 29(7):845-854.

18. Wolf MG, Hoefling M, Aponte-Santamaria C, Grubmuller H, & Groenhof G (2010) g_membed: Efficient insertion of a membrane protein into an equilibrated lipid bilayer with minimal perturbation. *J Comput Chem* 31(11):2169-2174.

19. Jorgensen WL, Chandrasekhar J, Madura JD, Impey RW, & Klein ML (1983) Comparison of Simple Potential Functions for Simulating Liquid Water. *J Chem Phys* 79(2):926-935.

20. Bussi G, Donadio D, & Parrinello M (2007) Canonical sampling through velocity rescaling. *J Chem Phys* 126(1):Artn 014101.

21. Berendsen HJC, Postma JPM, Vangunsteren WF, Dinola A, & Haak JR (1984) Molecular-Dynamics with Coupling to an External Bath. *J Chem Phys* 81(8):3684-3690.

22. Klauda JB*, et al.* (2010) Update of the CHARMM All-Atom Additive Force Field for Lipids: Validation on Six Lipid Types. *J Phys Chem B* 114(23):7830-7843.

23. Darden T, York D, & Pedersen L (1993) Particle Mesh Ewald - an N.Log(N) Method for Ewald Sums in Large Systems. *J Chem Phys* 98(12):10089-10092.

24. Foloppe N & MacKerell AD (2000) All-atom empirical force field for nucleic acids: I. Parameter optimization based on small molecule and condensed phase macromolecular target data. *J Comput Chem* 21(2):86-104.

25. Jo S, Kim T, Iyer VG, & Im W (2008) CHARMM-GUI: a web-based graphical user interface for CHARMM. *J Comput Chem* 29(11):1859-1865.

26. Zhu FQ, Tajkhorshid E, & Schulten K (2004) Collective diffusion model for water permeation through microscopic channels. *Phys Rev Lett* 93(22):Artn 224501.

27. Aponte-Santamaria C, Hub JS, & de Groot BL (2010) Dynamics and energetics of solute permeation through the Plasmodium falciparum aquaglyceroporin. *Phys Chem Chem Phys* 12(35):10246-10254.

28. Humphrey W, Dalke, A. and Schulten, K. (1996) VMD - Visual Molecular Dynamics. pp 33-38.

29. Hub JS, Winkler FK, Merrick M, & de Groot BL (2010) Potentials of mean force and permeabilities for carbon dioxide, ammonia, and water flux across a Rhesus protein channel and lipid membranes. *J Am Chem Soc* 132(38):13251-13263.

30. Hub JS, de Groot BL, & van der Spoel D (2010) g_wham-A Free Weighted Histogram Analysis Implementation Including Robust Error and Autocorrelation Estimates. *J Chem Theory Comput* 6(12):3713-3720.

31. Hub JS & de Groot BL (2008) Mechanism of selectivity in aquaporins and aquaglyceroporins. *Proc Natl Acad Sci U S A* 105(4):1198-1203.
